# Deficiency of vital organic nutrients in ecosystems limits brain development and fitness in wild fish

**DOI:** 10.1242/jeb.250914

**Published:** 2026-02-12

**Authors:** Libor Závorka, Johan Höjesjö, Stefan Auer, Benedikte Austad, Francesco Dionigi, Pernilla Hansson, Shaun S. Killen, Stefano Mari, Evelina Olsen, Matthias Pilecky, Kurt Pinter, Alexandra Polonyiová, Patrik Stehlík, Tileuzhan Smagul, Simon Vitecek, Mourine J. Yegon, Pavel Němec

**Affiliations:** ^1^ WasserCluster – Biologische Station Lunz, Inter-University Centre for Aquatic Ecosystem Research, Dr Carl-Kupelwieser Promenade 5, 3293 Lunz/See, Austria; ^2^ School of One Health, Biodiversity & Veterinary Medicine, College of Medical, Veterinary and Life Sciences, University of Glasgow, Glasgow, G61 1QH, UK; ^3^ Department of Biological and Environmental Sciences, University of Gothenburg, Medicinaregatan 7B, 413 90 Gothenburg, Sweden; ^4^ Institute of Hydrobiology and Aquatic Ecosystem Management, BOKU University, Gregor-Mendel-Straße 33, A-1180 Vienna, Austria; ^5^ Department of Zoology, Faculty of Science, Charles University, 128 44 Prague, Czech Republic; ^6^ Department of Functional and Evolutionary Ecology, University of Vienna, Djerassiplatz 1, 1030 Vienna, Austria; ^7^ University for Continuing Education Krems, Research Lab for Aquatic Ecosystem Research, Dr Karl-Dorrek-Straße 30, A-3500 Krems, Austria; ^8^ University of Innsbruck, Department of Ecology, Technikerstrasse 25, 6020 Innsbruck, Austria

**Keywords:** Omega-3, Anthropogenic environmental change, Freshwater food webs, Salmonid fishes, Cognitive ecology

## Abstract

Animals in aquatic ecosystems impacted by global changes often face reduced availability of vital organic compounds, such as long-chain omega-3 polyunsaturated fatty acids (n-3 LC-PUFA), which are essential for brain development and cognition. Cognitive skills are crucial for buffering the impacts of environmental stress on fitness, yet the link between the quality of diet and fitness-enhancing behaviours of individuals in food webs altered by global change remains unclear. We examined how dietary n-3 LC-PUFA affect brain development, social dominance and growth in territorial juvenile salmonids in a large-scale model of a natural pre-alpine stream. For this assessment, we used wild fish whose diet quality was estimated using stable isotope analysis, and hatchery-reared fish exposed to dietary treatments in a common-garden experiment. In both wild and common-garden experiment fish, diets low in n-3 LC-PUFA led to a decreased content of n-3 LC-PUFA in brain tissue but did not affect brain mass, morphology (i.e. mass of brain regions) or neuron numbers. Fish with lower brain n-3 LC-PUFA content exhibited reduced competitiveness in social interactions and suboptimal habitat use, resulting in slower somatic growth. Our findings indicate that the limited availability of n-3 LC-PUFA in aquatic food webs may impair the behavioural flexibility of top aquatic consumers, possibly with negative impacts on their capacity to maintain high fitness in ecosystems altered by environmental change.

## Teaser

**Summary*:*** Omega-3 LC-PUFA deficiency in aquatic ecosystems alters brain fatty acid composition in salmonids, reducing their cognitive capabilities and competitiveness in social interactions, highlighting the role of diet in fitness under environmental change.

## INTRODUCTION

The cognitive buffer hypothesis predicts that behavioural flexibility confers advantages in unpredictable and novel environments, improving the capacity of animals to cope with anthropogenic environmental changes (Sol et al., 2005). However, the benefits of high behavioural flexibility come with significant energetic and nutritional costs for the development and maintenance of neural systems (Pilecky et al., 2021; Heldstab et al., 2022). The brains of juvenile animals and animals with indeterminate neuronal growth such as fishes exhibit a high plasticity in response to environmental pressures (Ebbesson and Braithwaite, 2012; DePasquale et al., 2016). The plastic changes in brain development caused by environmental pressures such as elevated temperature (Závorka et al., 2020), high water turbidity (Pike et al., 2018) or reduced diet quality (Ishizaki et al., 2001; Koene et al., 2025) can influence cognitive performance and behaviour of fishes. However, it remains an open question how such laboratory measurements of brain and cognitive plasticity translate into behavioural flexibility in the natural environments and how brain plasticity might influence the capacity of fishes to maintain a high fitness in freshwater ecosystems altered by global changes in environment and climate.

One of the key nutrients for vertebrate brain development is long-chain omega-3 polyunsaturated fatty acids (n-3 LC-PUFA), which account for more than a third of the brain lipid mass and are crucial for signal transfer efficiency and neuronal plasticity (Pilecky et al., 2021). There are pronounced differences in the availability of n-3 LC-PUFA across food webs, with aquatic planktonic algae being the prime producer of n-3 LC-PUFA (Twining et al., 2016). Importantly, anthropogenic pressures such as climate change, habitat degradation, pollution and eutrophication have been shown to disrupt the production of n-3 LC-PUFA in aquatic food webs and their transfer within and across ecosystems (Závorka et al., 2023; Shipley et al., 2024). There is a risk that the paucity of dietary n-3 LC-PUFA in aquatic ecosystems will lead to maladaptive changes in neural traits of fishes, such as a reduction of brain size (Ishizaki et al., 2001), reduced neuron numbers (Kawakita et al., 2006) and lower cerebral content of n-3 LC-PUFA (Závorka et al., 2021). All of these neural traits, including brain size (Triki et al., 2022), neuronal number (Marhounová et al., 2019) and n-3 LC-PUFA content (Lund et al., 2014), have been consistently shown to be positively associated with cognition in fishes. Therefore, we posit that reduced availability of n-3 LC-PUFA in aquatic food webs may impair fishes' ability to cope with anthropogenic environmental changes due to direct effects of dietary n-3 LC-PUFA deprivation on neural traits and corresponding effects on cognition and behaviour. Testing this prediction requires simultaneous assessment of ecological factors and individual's fitness proxies (e.g. body growth rate), neural traits and cognitive performance in a natural context, at a level of scrutiny rarely undertaken.

The variation in the physical and social environment experienced by juvenile stream-dwelling salmonids, such as brown trout (*Salmo trutta*), provides an ideal system for investigating the impact of changing diet quality on neural traits, behavioural flexibility and fitness in an ecologically relevant context. Juvenile stream salmonids exhibit intra-specific variation in their dietary reliance on n-3 LC-PUFA-rich aquatic invertebrates versus n-3 LC-PUFA-deprived terrestrial invertebrates (Závorka et al., 2022). These fish also face an ongoing decrease in n-3 LC-PUFA availability as a result of factors such as rising temperatures (Hixson and Arts, 2016) and biodiversity degradation (Shipley et al., 2024). Juvenile brown trout are territorial animals that establish a linear social hierarchy when competing for patches of suitable micro-habitats in their nursery streams (Kalleberg, 1958; Bachman, 1984). The probability of dominance in social interactions among size-matched individuals is directly influenced by cognitive performance, such as perceiving contested habitat quality (Piccolo et al., 2014) or evaluating their opponent's social position through social eavesdropping (Johnsson and Åkerman, 1998; Johnsson et al., 2000; Koene et al., 2025). Dominant individuals in the social group occupy the most profitable habitats (Nakano, 1995) and benefit from their social position in terms of increased fitness indicators, such as higher somatic growth (Höjesjö et al., 2002). Therefore, habitat use, social hierarchy position and growth rate are suitable proxies for assessing the ecologically relevant outcomes of the dietary n-3 LC-PUFA effect on brain plasticity and cognition in wild trout.

Here, we investigated the effect of dietary n-3 LC-PUFA on neural traits critical for brain function, such as brain mass and mass of brain regions, neuron numbers and fatty acid content in juvenile brown trout. To do so, we used a combination of two complementary approaches: (1) by inferring to the diet quality of wild fish using stable isotope analysis and by conditioning juvenile brown trout to diets containing low and high amounts of n-3 LC-PUFA in a common-garden experiment; and (2) by testing at the individual level the link between neural traits and indicators of ecological performance, such as social dominance, habitat use and somatic growth rate, in large seminatural mesocosms simulating a pre-alpine stream habitat. We predicted that a limited dietary intake of n-3 LC-PUFA would negatively affect the development of the brain, leading to reduced ecological performance and fitness of juvenile trout.

## MATERIALS AND METHODS

The experimental work of this study consists of three major parts. First, the common-garden experiment, where we exposed brown trout, *Salmo trutta* Linnaeus 1758, from different populations to diets with high and low n-3 LC-PUFA content (see Table S1, Fig. S1). Second, a field survey of wild fish in streams, assessing the intake of aquatic prey naturally rich in n-3 LC-PUFA compared with terrestrial prey that is relatively poor in n-3 LC-PUFA (Závorka et al., 2022); this was achieved by analysing δ^13^C (an indicator of dietary reliance of fish on aquatic versus terrestrial resources) and δ^15^N (an indicator of trophic position) in muscle tissue of the fish. These first two parts of the experiment allowed us to test whether diet quality – either experimentally controlled or based on individual choice in the wild – influences the neural traits of the brain. Finally, in the third part of the experiment, we used a large-scale model of a natural pre-alpine stream to test the effect of brain neural traits on the behaviour and ecological performance of fish from both the common-garden experiment and the wild. All procedures in this study involving living animals were permitted under licence from the Austrian Federal Ministry of Education, Science and Research (licence GZ: 2023-0.053.856), and complied with all regulations valid in Austria and the EU.

### Common-garden experiment

At the end of October 2022, juvenile brown trout (10 months post-hatching) from four hatcheries maintaining genetically diverse brood stocks based on wild-caught fish from the Danube catchment (Lercetau-Köhler et al., 2013; Schenekar and Weiss, 2017) were transferred to the fish husbandry facility of WasserCluster Lunz (gps: 47.8544136N, 15.0679575E). In addition, we collected wild fish of the same age (i.e. 0+) from River Ybbs by electrofishing (EFKO 1500, Germany) and included them in the common-garden experiment (see Table S1). The common-garden experiment started with 70 individuals from each population. We chose the majority of fish from the hatchery rather than of wild origin for the common-garden experiment, because the prolonged captivity required for the dietary treatment can have negative effects on the behaviour, physiology and survival of wild brown trout (Závorka et al., 2019; Johnsson and Näslund, 2018). Using a wild population in the common-garden experiment allowed us to control for a potential difference in the response of hatchery-reared and wild fishes to dietary intake of n-3 LC-PUFA (e.g. Betancor et al., 2016). The fish were kept in 500 l holding tanks with flow through water (2 l min^−1^; complete water change ∼6 times a day) fed by a nearby spring. The temperature in the tanks corresponded to the ambient temperature of the source spring water, which had small daily fluctuations but varied across seasons from ∼4°C in January/February to ∼14°C in June/August. The light regime in the facility was set to the local time of sunrise and sunset, adjusted in 7 day intervals. Upon arrival, fish were distributed according to their origin among 10 holding tanks with 32±2 individuals in each tank (i.e. fish from different hatcheries were not mixed together and each population occupied two holding tanks). Fish were fed daily to apparent satiation via feeding belts that slowly delivered feed pellets between 09.00 h and 16.00 h. The food dose was adjusted according to the amount of uneaten feed remaining at the bottom of the holding tank. During the first 2 weeks, the hatchery fish were fed the feeds of their hatcheries of origin, while the wild fish were fed a mixture of feeds from the experimental diets. From 22 November 2022, all individuals were fed exclusively on one of the two experimental diets (Aqua Garant, Pöchlarn, Austria), so each tank per population received a different diet. We used two isocaloric diets, of near-identical nutritional value, but one diet was high in n-3 LC-PUFA and the other was limited in n-3 LC-PUFA (low). For details of the biochemical composition of diets and their resemblance to the biochemical composition of natural prey of brown trout, see Závorka et al. (2021) and Tables S2 and S3.

On 15 February 2023, all fish were tagged with 12 mm PIT-tags (Biomark, Rahway, NJ, USA) and body mass and fork length were measured to the nearest 0.1 g and 1 mm, respectively. Before tagging and body size measurements (in this and all other such events during the study), fish were anaesthetized by individually submerging them in a well-aerated water solution containing 0.5 ml l^−1^ 2-phenoxyethanol until they lost equilibrium and showed a substantial reduction in opercular respiratory movements. After the procedure, the fish were transferred to the recovery tank with fresh, well-aerated water until they resumed active ventilation, equilibrium and normal swimming behaviour. After tagging, individuals were redistributed among the 10 holding tanks in groups of 32±2 size-matched individuals, forming a mix of populations while maintaining their original dietary treatments (i.e. as of 22 November 2022). From mid-May on, a subset of 24 individuals from each population was successively used for observations in the stream mesocosms (Table S1, Fig. S1). In total, we tested nine populations in the study (five from the common-garden experiment and four from the wild), which means that there was approximately a 3 month difference between the first and last batches of fish observed in stream mesocosms. For more details on the timing of experimental steps, see Fig. S1. Survival during the common-garden experiment was 90%. The rest of the individuals were used in another study (Mari et al., 2025) and then euthanized as a humane endpoint by overdose of anaesthetics (i.e. individuals were submerged in a well-aerated 10°C water solution containing 2 ml l^−1^ 2-phenoxyethanol for 30 min or until they were no longer responsive to physical stimuli) and subsequent transection of the spinal cord and decapitation. Individuals used in the stream mesocosms were acclimatized to natural prey in their holding tanks for a period of 10 days before the transfer, by adding live benthic macroinvertebrates collected by kick sampling in a nearby stream to the holding tanks (Seebach, 47.8523772N, 15.0651542E; see Table S4 for taxonomical composition of the prey). During the first 5 days, macroinvertebrates were provided along with the experimental feed, while during the remaining 5 days, the experimental feed was completely withheld, and the fish were fed only on macroinvertebrates.

### Field survey

Wild individuals of juvenile brown trout (age 0+ and 1+) from four different source populations (Table S1) were caught by electrofishing and transferred from their streams of origin to the husbandry facility (*N*=30 per population). Fish were tagged with 12 mm PIT-tags (Biomark) upon arrival and kept in the same holding tank conditions as described for the common-garden experiment for 10 days, after which they were fed live benthic macroinvertebrates before being transferred to the stream mesocosm facility. Samples of potential prey of brown trout were collected at each sampling site as a baseline for stable isotope analysis (see below). Benthic macroinvertebrates were collected using kick sampling, and terrestrial macroinvertebrates were collected by hand picking and dragging a net over the canopy surrounding the streams. Samples collected at each site were stored alive in an icebox and, after determination in the laboratory, kept frozen at −70°C until further processing.

### Behavioural measurements in stream mesocosms

The stream mesocosms used in this study are two 40 m long gravel-bottom channels simulating the natural habitat of a small pre-alpine stream (Auer et al., 2023). Each of the two channels was split transversally using a fence with a mesh size of ∼0.5 cm^2^ into four equivalent experimental enclosures (*N*=8). Each enclosure was 7 m long and 1.5 m wide and they were separated from each other by 2 m long buffer zones (see Supplementary Materials and Methods, ‘Further description of the stream mesocosms’ and Figs S2–S4 and Table S4). The cross-sectional profile of each stream mesocosm enclosure included a deep side (1 m wide) and a shallow side (0.5 m wide). The water discharge through each channel was constant at 25 l s^−1^, and the average water temperature ranged from 8°C to 12°C throughout the study (Table S1). The water level was adjusted by wooden panels placed at the downstream end of each enclosure. During high water levels, the water depth was ∼30 cm in the deep part and ∼15 cm in the shallow part of each section, with an average water flow of ∼7 cm s^−1^. During low water levels, the water depth was ∼15 cm in the deep part and ∼0 cm in the shallow part, with an average water flow of ∼16 cm s^−1^. Longitudinally, each section was divided into three equally long habitats differing in the grain size of the bottom substrate: the most upstream part had the largest grain size (pebbles and gravel), the intermediate part contained gravel and the most downstream part contained coarse sand. The deep part of each section also contained four red clay flowerpots (25 cm diameter) that were half-buried in the substrate, with the openings facing downstream. These shelters were placed at positions 1, 3, 4 and 6 m within the section (measured from downstream to upstream). The most upstream pot was enriched with a 0.5 m long piece of submerged wood anchored to the flowerpot. Buffer zones between the sections contained pebbles enriched with large boulders and pieces of submerged wood (Fig. S2). The acclimation pool was covered with mesh, and each mesocosm channel was fenced with an electric fence to prevent access by predators (e.g. piscivorous birds and mammals).

In early May 2023, before the start of the first experimental round, each enclosure of the mesocosms was inoculated with benthic macroinvertebrates collected from a nearby stream (see Table S4 for taxonomic composition). Macroinvertebrates for each section were collected by a standardized kick sampling (Brown and Brussock, 1991) from an area ∼7.5 m^2^. We stocked approximately 60,000 macroinvertebrates in each enclosure, resulting in a density of ∼5000–6000 individuals per m^2^, which corresponds to the lower end of natural macroinvertebrate density in a pre-alpine stream (Leitner et al., 2015). This density was maintained throughout the study. Qualitative checks of the abundance of macroinvertebrates in the experimental sections were done at regular 3 week intervals, and restocking took place at the end of June and beginning of August to maintain similar prey abundance throughout the study. In total, over the duration of the experiment from May until August, each enclosure was supplied by ∼150,000 individual macroinvertebrates. Substrate in the buffer zones was disturbed before each experimental round and at the day 6 of each round to release accumulated macroinvertebrates to the experimental enclosures.

Batches of experimental individuals were successively transported to the stream mesocosm facility 7±2 days before the start of the experiment and released into separate acclimation pools to acclimatize to the water and habitat quality of the mesocosms (Supplementary Materials and Methods, ‘Further description of the stream mesocosms’, Fig. S2). After the acclimation period, individuals were collected from the acclimation pool using electrofishing, measured for their body mass (to the nearest 0.1 g) and fork length (to the nearest 1 mm), and distributed among one of eight experimental enclosures. Each enclosure hosted six size-matched juvenile brown trout, always from the same population of origin, which corresponded to a density of 0.6 fish per m^2^, which falls within the medium range of natural density of brown trout in nursery streams (Bohlin et al., 2002; Pinter et al., 2019). Individuals from wild populations were distributed based on their body size (i.e. fish were size matched and from the same population within each enclosure), whereas individuals from the populations exposed to the common-garden experiment were stocked within the given enclosure to be size matched and according to their dietary treatment, with three individuals fed the n-3 LC-PUFA-rich diet and three individuals fed the n-3 LC-PUFA-poor diet and all individuals in the enclosure being from the same population. The sex of individuals was determined after the experiment (see below) and thus could not be considered when distributing individuals among the experimental enclosures.

The period of behavioural observation in each round of the stream mesocosm experiment lasted 10 days (Table S1). To simulate the fluctuation of water level typical for pre-alpine streams (e.g. Caine, 1992), each round included an initial period with high water levels from day 1 to day 4, a low water level period from day 5 to day 6, followed by a high water level period from day 7 to day 10. We considered behavioural data in the stream mesocosm enclosures only up to day 7, because on this day, one individual was removed from each enclosure by electrofishing to be used in another study (B.A., L.Z., S.M., S.A. and Johan Höjesjö, submitted). The longitudinal position of individual trout in the stream was determined using active RFID telemetry with a portable antenna (HPR Plus reader with BP Plus Portable Antenna, Biomark). Portable antenna screening was always conducted in the direction from the down- to up-stream end of the enclosure.

The identity of the dominant individual in each enclosure was identified using a combination behavioural assessment based on video recordings [obtained with video cameras (RLC-810A, Reolink, Reolink, China) equipped with additional infra-red spotlights (see Supplementary Materials and Methods, ‘Further description of the stream mesocosms’ and Figs S2–S4 and Table S4)] and PIT-tag identification through stationary RFID telemetry with two stationary antennas per enclosure (Multiple Antenna PIT-tag reader, Oregon RFID, Portland, OR, USA). An individual was classified as dominant when all of the following criteria were met: (i) the dominant individual displayed pale skin coloration compared with subdominant individuals (O'Connor et al., 1999); (ii) the dominant individual received no aggressive attacks from the rest of the group, but actively attacked and chased other individuals (Höjesjö et al., 2002); and (iii) the dominant individual occupied a central position in the section, allowing control over the most profitable upstream microhabitat (Kalleberg, 1958). We were able to clearly determine the dominant individual in 35 out of 36 experimental enclosures. While we could not determine the dominant individual in one enclosure because of a technical failure causing a missing video record, social dominance appeared to be clearly established in all enclosures at the latest by day 7 of the experiment (Fig. S3). Given the camera point of view (see Fig. S4), we were not able to distinguish fine-scale interactions, such as aggressive display among individuals, but aggressive attacks and the social dominance status of individuals were clearly visible (see Movie 1 for a sample video). We then linked the behavioural identification of the dominant individual to the PIT-tag ID through the time stamp of the video recordings when this fish passed through one of the two stationary RFID antennas, and matched the video with time stamped PIT-tag ID detections from the corresponding RFID antenna. Each dominant individual was determined based on multiple independent PIT-tag ID detections.

Finally, at the end of each round on day 10, all individuals were collected from the stream mesocosm by electrofishing, and their body mass and fork length were measured again. The individuals were then euthanized with an overdose of anaesthetic (2 ml l^−1^ of 2-phenoxyethanol), followed by spinal cord transection. Samples of dorsal muscle and brain tissue were collected for biochemical and histological analysis, and a fin clip was taken for genetic sex determination. The specific growth rate (SGR) of individuals was calculated using the equation from Brett and Groves (1979):
(1)

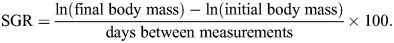
The SGR during the mesocosm experiment was determined based on the initial and final body mass measured at the start and end of the stream mesocosm experiment. Similarly, the SGR of fish from the common-garden experiment during the feeding treatment preceding the stream mesocosm experiment was calculated using the initial mass recorded on 15 February 2023, and the mass at the beginning of the stream mesocosm experiment.

### Brain dissection

Immediately after euthanasia, we opened the fish skull and collected ∼80% of fresh tissue from the left optic tectum by a midsagittal cut for fatty acid analysis. We had to open the skull and extract fresh tissue from the left optic tectum within 5–10 min to prevent degradation. Therefore, we could not extract 100% of the tissue, because the precision needed for such extraction was not possible in the given time frame without the risk of damaging other brain parts. This fresh tissue was stored on dry ice and then at −70°C until further processing. The whole head with partially exposed brain was then fixed by immersion in 4% phosphate-buffered paraformaldehyde solution. After 24 h, the brains were dissected, rinsed in phosphate buffer (pH 7.4) to remove residual paraformaldehyde, and then stored in antifreeze solution (30% glycerol, 30% ethylene glycol, 40% phosphate buffer) at −20°C until further processing.

### Determination of total cell and neuron number

Brains were divided into four parts, namely the telencephalon, optic tectum, cerebellum and ‘rest of the brain’, comprising the diencephalon, tegmentum and medulla oblongata. The remaining ∼20% of the left optic tectum was removed and discarded, and we further analysed only the right optic tectum and multiplied the results by two to get comparable data with the other brain parts. During the dissection of the brain, we removed the olfactory bulb because this part of the brain is prone to damage during the dissection. Brain parts were weighed to the nearest 0.00001 g using a Kern ABT 120-5DNM analytical balance (Kern & Sohn GmbH, Balingen-Frommern, Germany). Total brain mass was calculated as the sum of all measured brain parts. Cell and neuron numbers in each of the above-mentioned brain parts were assessed using a modified isotropic fractionator technique (Marhounová et al., 2019). Dissected brain parts were homogenized in 40 mmol l^−1^ sodium citrate with 1% Triton X-100 using Tenbroeck tissue grinders (Wheaton, Millville, NY, USA) to obtain a suspension of free cell nuclei. The fluorescent DNA marker DAPI was added (0.5 mg l^−1^) to stain the nuclei, the homogenate was adjusted to the defined volume, and the mixture was kept homogeneous by agitation. The total number of cells was estimated by counting at least six aliquots of 10 µl using a Neubauer improved counting chamber (BDH, Dagenham, Essex, UK) with an Olympus BX51 microscope equipped with epifluorescence and appropriate filter settings; additional aliquots were counted when needed to reach a coefficient of variation among counts ≤0.05. Care was taken to exclude erythrocytes from counts of the brain cells (erythrocytes were easily recognizable because of the typical ellipsoid shape of their nuclei). The mean of all the samples for the given structure (number of nuclei per 0.1 µl) was then multiplied by 10,000 to obtain the number in 1 ml and by the total volume of the homogenate in ml to derive the actual number of cells in the structure.

The proportion of neurons was determined by immunocytochemical detection of the neuronal nuclear marker NeuN. This neuron-specific protein was detected by an anti-NeuN rabbit polyclonal antibody (lot: 4000672; Merck Millipore; dilution 1:800). The binding sites of the primary antibody were revealed by a secondary anti-rabbit antibody conjugated with Alexa Fluor 594 (Life Technologies, Carlsbad, CA, USA; dilution 1:400). An electronic haematologic counter (Alchem Grupa, Torun, Poland) was used to count the proportion of double-labelled nuclei in the Neubauer chamber. At least 500 nuclei were examined for each sample. The percentage of immunopositive nuclei was then applied to the previously determined cell count to derive the absolute number of neurons and non-neuronal cells. The number of cells and neurons was assessed in a subsample of 70 individuals from the common-garden experiment and 39 wild individuals, evenly distributed across dietary treatments, sexes and populations of origin.

### Fatty acids and stable isotope analysis

We used bulk tissue analyses of δ^13^C and δ^15^N of trout muscle tissue and potential prey sources to estimate the reliance of wild brown trout on terrestrial and aquatic prey in their stream of origin. Fresh muscle tissue was sampled from the dorsal region between the dorsal fin and the lateral line, with skin and bones removed during sampling. The tissue was stored in the same way as the brain samples (described above). Freeze-dried and homogenized samples of trout muscle tissue and prey sources were prepared following the procedure described by Bessey and Vanderklift (2014). Isotope ratios are reported relative to the international Vienna PeeDee Belemnite carbonate (δ^13^C) standard and air (δ^15^N). δ^13^C and δ^15^N values were mathematically corrected for the basal resource values at each sampling site (Fedorčák et al., 2025) to ensure comparability across streams of fish origin. Higher δ^13^C indicated a reliance on n-3 LC-PUFA-poor terrestrial prey (Závorka et al., 2022); correspondingly, the local δ^13^C of terrestrial prey in the four sampled streams was, on average, 2.3‰ higher than that of aquatic prey. δ^15^N values served as an indicator of the trophic position of individuals, with higher δ^15^N indicating a higher trophic position (Bunn et al., 2013).

Fatty acids were extracted and analysed from freeze-dried samples (3–10 mg dry mass) that were homogenized, sonicated and vortexed (3 times) in a chloroform–methanol (2:1) mixture, following Pilecky et al. (2023). Total lipid mass ratios were determined by gravimetry. Fatty acids were derivatized to obtain fatty acid methyl esters (FAME) using toluene and 1% sulphuric acid in methanol (incubated for 2 h at 70°C). FAME were separated on and quantified using a gas chromatograph (Thermo Scientific TRACE GC Ultra) equipped with a flame ionization detector (FID) and an Agilent HP-88 column (100 m, 25 mm i.d., 0.2 μm film thickness). Quantification of fatty acids was performed by comparison with a known concentration of the internal standard using Chromeleon 7. All the fatty acid values reported in this study are relative percentage of fatty acids with respect to total FAME.

### Sex determination

Fin clips were used as a source of nuclear DNA for sex determination following a modified duplex PCR protocol. Following the manufacturer's instructions, a NucleoSpin™ Tissue kit (Macherey-Nagl) was used to extract genomic DNA from adipose fin clips. Quality was controlled with spectrophotometry (NanoDrop, ThermoFisher Scientific), and quantification was conducted fluorometrically (Qubit 2.0, ThermoFisher Scientific), before dilution to 20 ng μl^−1^. Duplex PCR amplified the male chromosome gene, *sdY*, with *18S* as a positive amplification control, and agarose gel (2%) electrophoresis was used to visualize the resulting products (Koene et al., 2025).

### Data processing and analysis

All analyses were conducted in R v.4.4.2 ‘Pile of Leaves’ (http://www.R-project.org/). See Table 1 for the structure of models testing the effect of diet quality on brain neural traits and the effect of the neural traits on the ecological performance of fish in the stream mesocosms. The effect of diet quality on neural traits was tested by linear models (LM) and controlled for the fish population, sex and fork length as co-variables. The effect of neural traits on the probability of becoming dominant among the group of conspecifics in the stream mesocosms was evaluated by generalized linear mixed models (GLMM) and generalized linear models (GLM) for data with a binomial distribution (1=dominant, 0=subdominant). The effect of brain traits and social dominance on specific growth rate of body mass (SGR mass) was tested by linear mixed models (LMM). Initial models for dominance and SGR mass included fish origin (i.e. common-garden or wild) and the interaction between neural trait and fish origin, sex, body size rank and population as a random factor. Non-significant interaction terms and random factors with predicted variance near zero were removed from the final models. To assess traits of an individual relative to conspecifics from the same experimental enclosure in the stream mesocosm, we used rank instead of the absolute value of the trait. This approach yielded the best model fit and distribution of residuals for our data compared with other normalization methods. It allowed us to assess the performance of individuals relative to that of conspecifics from the same population, length of dietary treatment exposure, facing the same environmental conditions such as water temperature, prey availability, weather, position of the enclosure within the stream mesocosm and distribution of phenotypic traits within the social group. For example, body size rank was based on the fork length of an individual at the beginning of stream mesocosm experiment relative to that of the other fish within the same enclosure of the stream mesocosm ranging from 1 (for the largest) to 6 (for the smallest) individuals in the enclosure. Only enclosures with data for the given trait available for at least 4 individuals were included. The same ranking logic was used to establish the n-3 LC-PUFA rank, n-6 PUFA rank, telencephalon mass rank and whole-brain mass rank). The mass of telencephalon was closely related to the total mass of the brain (see Tables S8 and S9). Therefore, telencephalon mass rank was based on residuals from a log–log linear regression between the mass of the telencephalon and the mass of the whole brain minus the mass of the telencephalon. We did not analyse the effect of neuron number on individual performance in the stream mesocosms because neuronal counts were available for only 109 of the 216 individuals used in the experiment. Consequently, almost all enclosures failed to meet the criterion that at least four out of six individuals had data available for inclusion in the analysis.

**Table 1.** Summary of final models reported in Results

Model group	Model type	Subset	Response variables	Explanatory variables
Brain quality	LM	Wild	SFA, MUFA, n-3 LC-PUFA, n-6 PUFA, BRM	δ13C + δ15N + sex + FL + population
		Common-garden		diet + sex + FL + population
		Wild	TelM, CbM, OTM, RoBM, BRN	δ13C + δ15N + sex + FL + BRM + population
		Common-garden		diet + sex + FL + BRM + population
		Wild	TelN, CbN, OTN, RoBN	δ13C + δ15N + sex + FL + TelM or CbM or OTM or RoBM + population
		Common-garden		Diet + sex + FL + TelM or CbM or OTM or RoBM + population
Social dominance	GLMM (binomial)	All individuals	Dominant in the group (1 or 0)	n-3 LC-PUFA rank, or n-6 PUFA rank, or rTelM rank, or rBRN rank * origin + sex + Body size rank + (1| population)
Growth	LMM	All individuals	SGR mass	Dominant * origin + sex + Body size rank + (1| population)
		Subdominant		n-3 LC-PUFA rank, or n-6 PUFA rank, or rTelM rank, or rBRN rank * origin + sex + Body size rank + (1| population)
Habitat use		All individuals	Longitudinal position in the mesocosm	Dominant * Day + sex + Body size rank + origin + (1|Fish ID) + (1| population)
		Subdominant		n-3 LC-PUFA rank, or n-6 PUFA rank, or rTelM rank, or rBRN rank * Day + origin + sex + Body size rank + (1|Fish ID) + (1| population)

Diet: common-garden dietary treatment (category with two levels: high or low n-3 LC-PUFA diet); FL: fork length at the end of experiment; BRM, TelM, CbM, OTM, RoBM: mass of the whole brain, telencephalon, cerebellum optic tectum and the rest of the brain, respectively; BRN, TelN, CbN, OTN, RoBN: number of neurons in the whole brain, telencephalon, cerebellum optic tectum and the rest of the brain, respectively; SFA, MUFA, n-6 PUFA, n-3 LC-PUFA: relative content (%) of saturated fatty acids, monounsaturated fatty acids, omega-6 polyunsaturated fatty acids and omega-3 long-chain polyunsaturated fatty acids, respectively; δ^13^C and δ^15^N: muscle isotopic values adjusted for the stream baseline; sex (category with two levels: male or female); origin (category with two levels: common-garden or wild); population (category with: nine levels, four in wild fish and five in common-garden fish); SGR mass: specific growth rate of body mass. LM, linear models; GLMM, generalized linear mixed models; LMM, linear mixed models.

The significance of the final models was evaluated using ANOVA tables with Type II and III sum of squares for models without and with significant interactions, respectively. Deviations from the assumptions of the models were diagnosed by visual inspection of the distribution of model residuals. The assumptions of the models were satisfactorily met in all cases. Differences among groups were analysed using Tukey's HSD *post hoc* test. *P*-values of the models were corrected for multiple comparison using the Bonferroni correction, treating all models based on the same subset, structure of explanatory variables and type of response variable as multiple comparison (Table 1).

## RESULTS

### Effect of dietary intake of n-3 LC-PUFA on neural traits

**Fig. 1.**
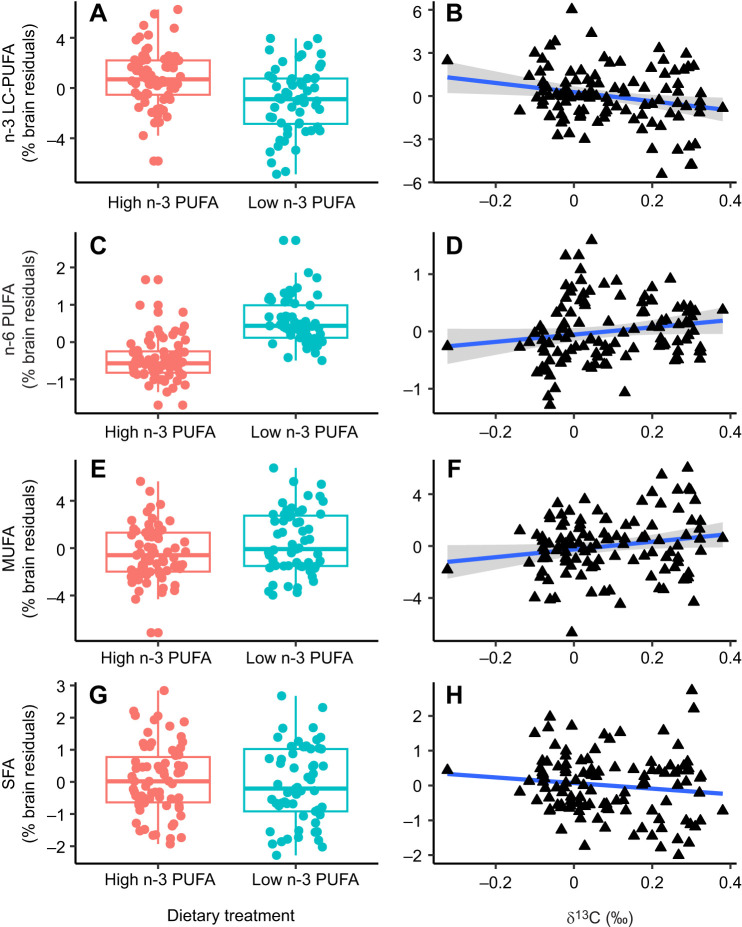
**Effect of diet quality on the fatty acid content of the trout brain.** Percentage of long-chain omega-3 polyunsaturated fatty acids (n-3 LC-PUFA) (A,B), omega-6 polyunsaturated fatty acids (n-6 PUFA) (C,D), monounsaturated fatty acids (MUFA) (E,F) and saturated fatty acids (SFA) (G,H) in brain residuals. Boxplots on the left illustrate differences in fatty acid content (high, red; low, green) among treatment groups in the common-garden experiment (*N*=57). The central lines represent the median, the box limits correspond to the 25th and 75th percentiles, and the whiskers extend to the 95th percentiles. Filled circles represent individual data points. Scatter plots with regression lines on the right show the correlation between δ^13^C of muscle tissue (corrected for stream baseline) and fatty acid content in the fish brain (*N*=112 for all plots except B, where *N*=111). Note that higher δ^13^C indicates a reliance on n-3 LC-PUFA-poor terrestrial prey. Shaded areas indicate s.e.m. for statistically significant relationships. *y*-axes represent residuals from a linear model controlled for population, sex and body size (i.e. fork length).

In the common-garden experiment, the diet rich in n-3 LC-PUFA led to an increase of n-3 LC-PUFA (Table 2, Fig. 1A) and a decrease of n-6 PUFA (Table 2, Fig. 1C) content of trout brain, but had no effect on monounsaturated fatty acid (MUFA) and saturated fatty acid (SFA) content (Table 2, Fig. 1E,G). Similar to the fish from the common-garden experiment, we found that wild fish consuming a high proportion of aquatic prey (rich in n-3 LC-PUFA) had higher n-3 LC-PUFA (Table 2, Fig. 1B) and lower n-6 PUFA (Table 2, Fig. 1D) content in their brain compared with conspecifics consuming more terrestrial prey. A higher intake of aquatic prey also led to a decrease of MUFA in the brain of wild brown trout (Table 2, Fig. 1F), but no effect of diet quality on SFA content in the brain of wild fish was observed (Table 2, Fig. 1H). There was no significant effect of diet quality on brain mass, relative mass of brain regions and neuron numbers of fish from the common-garden experiment or from the wild (Table 2; Tables S5–S10). We found no effect of trophic position (i.e. δ^15^N) on any measured neural trait of wild fish (Tables S6, S8 and S10).

**Table 2.** Effect of dietary intake of n-3 LC-PUFA on fatty acid composition, and mass and neuronal number of the whole brain and telencephalon

Subset	Dependent variable	*N*	*F*	*P*
Common-garden	n-3 LC-PUFA	126	16.88	**<0.0001**
	n-6 PUFA	126	94.51	**<0.0001**
	MUFA	126	4.08	0.0456
	SFA	126	0.69	0.4073
Wild	n-3 LC-PUFA	110	19.07	**<0.0001**
	n-6 PUFA	111	9.18	**0.0031**
	MUFA	111	11.53	**0.001**
	SFA	111	5.16	0.0252
	Brain mass	133	1.21	0.2731
Common-garden	TelM	133	0.70	0.4052
	OTM	133	3.49	0.0641
	CbM	133	0.59	0.5862
	RoBM	133	4.87	0.0294
Wild	Brain mass	119	2.48	0.1185
	TelM	119	0.54	0.4639
	OTM	119	1.05	0.3070
	CbM	119	2.23	0.1382
	RoBM	119	0.00	0.9988
Common-garden	Brain neurons	68	1.11	0.2954
	TelN	70	0.09	0.7635
	OTN	70	0.74	0.3923
	CbN	70	1.02	0.3169
	RoBN	70	1.60	0.2106
Wild	Brain neurons	39	0.27	0.6050
	TelN	39	1.46	0.2364
	OTN	39	1.97	0.5019
	CbN	39	0.51	0.4808
	RoBN	39	1.21	0.2790

For n-3 LC-PUFA, the explanatory variable for common-garden fish is diet, and that for wild fish is δ^13^C. Bold *P*-values are significant after Bonferroni correction for multiple hypothesis testing.

Population of origin and fork length had a significant effect on the content of all tested fatty acid groups in both wild and common-garden fish, with the exception that population of origin had no significant effect on SFA in wild individuals, and fork length had no significant effect on SFA and MUFA in common-garden fish (for details, see Tables S5 and S6). Population of origin had a significant effect on the mass of the telencephalon and the whole brain in individuals from the common-garden experiment, and on the mass of the optic tectum in wild individuals. Fork length was positively correlated with whole-brain mass, and the mass of individual brain regions was positively correlated with whole-brain mass in all models (for details, see Tables S7 and S8). Population of origin had no significant effect on neuronal number in either wild or common-garden fish. Neuronal number was predicted by the mass of the corresponding brain region, but this effect was significant only for the ‘rest of the brain’ in both wild and common-garden fish (for details, see Tables S9 and S10). Sex of individuals had no significant effect on any measured neural trait of wild and common-garden fish (for details, see Tables S5–S10).

### Effect of neural traits on social dominance, somatic growth and habitat use

**Fig. 2.**
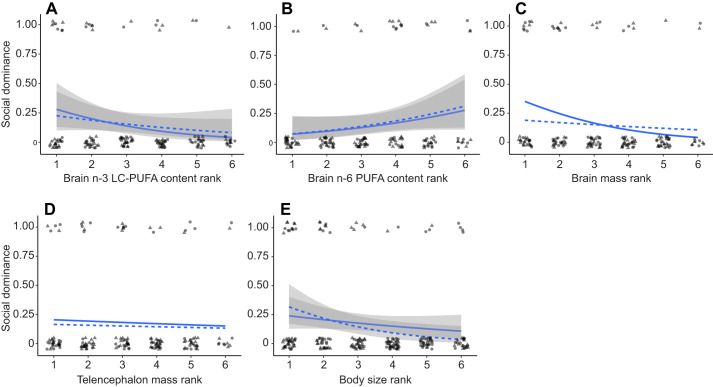
**Effect of neural traits on social dominance.** Logistic regression lines representing the relationship between brain trait values relative to those of other individuals in the enclosure and the probability of becoming dominant within a group of conspecifics. (A) Brain content of n-3 LC-PUFA, (B) brain content of n-6 PUFA, (C) whole-brain mass, (D) telencephalon mass and (E) body size. Solid lines represent relationships for fish from the common-garden experiment, while dashed lines represent relationships for wild fish. Data points for individuals from the common-garden experiment (*N*=75 for A,B; *N*=83 for C,D; *N*=93 for E) and the wild (*N*=74 for A,B; *N*=76 for C,D; *N*=82 for E) are depicted as circles and triangles, respectively. Shaded areas indicate s.e.m. for statistically significant relationships.

Trout with higher n-3 LC-PUFA and lower n-6 PUFA content in their brains were more likely to socially dominate the group in the experimental enclosure of the stream mesocosm. This effect remained significant even after controlling for body size of individuals (Table 3, Fig. 2A,B; Tables S11 and S12). In contrast, brain mass and telencephalon mass, after controlling for individual body size, showed no effect on social dominance (Table 3, Fig. 2C,D; Tables S11 and S12).

**Table 3.** Effect of brain traits on social dominance and somatic growth of individuals in stream mesocosms

Dependent variable	Explanatory variable	*N*	LR Chisq	*P*
Dominance	n-3 LC-PUFA rank	149	7.10	**0.0077**
	n-6 PUFA rank	149	5.62	0.0177
	Brain mass rank	159	0.56	0.4531
	TelM rank	159	0.23	0.6311
	Body size rank	159	15.40	**<0.0001**
SGR mass	Dominance	174	6.53	**0.0106**
SGR mass of subdominant	n-3 LC-PUFA rank	119	0.73	0.3934
	n-6 PUFA rank: origin	119	7.61	**0.0058**
	*n-6 PUFA rank (common-garden)*	*57*	*10.61*	***0.0011***
	*n-6 PUFA rank (wild)*	*62*	*0.36*	*0.5484*
	Brain mass rank	122	8.78	**0.0030**
	TelM rank	122	2.06	0.1508

LR Chisq, likelihood ratio chi-square test. Bold *P*-values are significant after Bonferroni adjustment for multiple comparisons. The rows in italics indicate the results of the models that break down the significant interaction between n-6 PUFA rank and origin. These results show a significant relationship between the SGR mass of subdominant individuals and n-6 PUFA rank for common-garden fish, but not for wild fish.

**Fig. 3.**
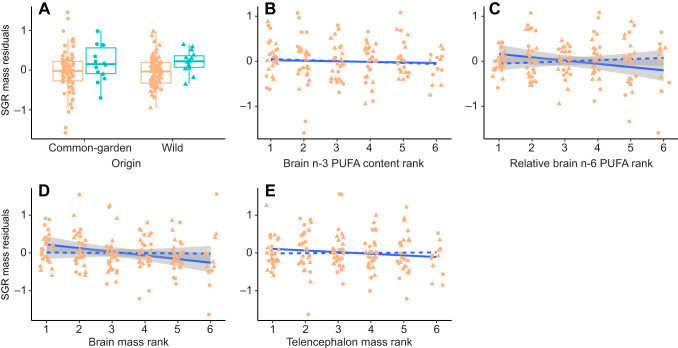
**Effect of brain traits on the specific growth rate of body mass in dominant individuals and the rest of the group in the stream flume mesocosms.** (A) Boxplots illustrating differences in the specific growth rate of body mass (SGR mass) between dominant (green; *N*=28) and subdominant (orange; *N*=146) individuals from the common-garden experiment (*N*=89) and the wild (*N*=85). (B–E) Scatter plots with regression lines showing the correlation between SGR mass and neural trait values relative to other individuals in the enclosure. Solid lines represent relationships for experimental fish from the common-garden experiment, while dashed lines represent relationships for wild fish. Data points for individuals from the common-garden experiment (*N*=57 for B,C; *N*=60 for D,E) and the wild (*N*=62 for B,C; *N*=62 for D,E) are depicted as circles and triangles, respectively. Shaded areas indicate s.e.m. for statistically significant relationships.

The dominant individuals clearly benefited from their position within the social group by having a faster somatic growth rate (Table 3, Fig. 3A). In addition, subdominant trout with greater brain mass exhibited faster growth compared with other subdominant individuals in the group (Table 3, Fig. 3D). The higher content of n-6 PUFA in the brain was associated with faster somatic growth among subdominant individuals, but only in the fish from the common-garden experiment and not in those brought directly from the wild (Table 3, Fig. 3C). The growth rate of subdominant individuals was not related to the n-3 LC-PUFA content of their brain (Table 3, Fig. 3B) or to the mass of the telencephalon (Table 3, Fig. 3E). We found no association between neural traits and somatic growth rate during the common-garden experiment preceding the stream mesocosm experiment (Table S13).

Telemetry data showed that dominant individuals were detected farther downstream compared with the group average, particularly on day 7 (Table 4; Fig. S5). Similarly, subdominant individuals with higher n-3 LC-PUFA content in the brain were detected farther downstream, particularly on day 7 (Table 4; Fig. S6). Subdominant individuals with higher n-6 PUFA content in the brain were also detected farther downstream, particularly on day 4 (Table 4; Fig. S7). Brain mass and telencephalon mass had no effect on the position of subdominant individuals in the stream mesocosm (Table 4).

**Table 4.** Effect of behavioural and brain traits on the position of individuals in the stream mesocosms

Dependent variable	Explanatory variable	*N*	LR Chisq	*P*
Stream position	Dominance	2054	0.54	0.4616
	Dominance: Day		11.83	**0.0371**
Stream position of subdominant	n-3 LC-PUFA rank	1527	0.09	0.7596
	n-3 LC-PUFA rank: Day		33.84	**<0.0001**
	n-6 PUFA rank	1527	1.46	0.2268
	n-6 PUFA rank: Day		14.78	**0.0113**
	Brain mass rank	1564	0.55	0.4548
	TelM rank	1525	0.40	0.5259

Bold *P*-values are significant after Bonferroni adjustment for multiple comparisons.

## DISCUSSION

Our study demonstrates a clear positive association between dietary intake of n-3 LC-PUFA and the probability of attaining social dominance within a group of conspecifics, as well as the associated benefit of faster somatic growth in territorial juvenile fish. The positive effect of diet quality on the behavioural performance of individuals appeared to be directly linked with higher n-3 LC-PUFA and lower n-6 PUFA content in the fish brain, but not with brain size, the size of its regions or neuron number. We showed that the observed patterns linking diet, neural traits, behaviour and the fitness proxy (i.e. somatic growth) in near-natural habitat are consistent in both wild fish and fish exposed to high and low n-3 LC-PUFA diets in a common-garden experiment. These results strongly suggest that the dietary intake of n-3 LC-PUFA-rich prey is essential for optimal brain function and fitness-enhancing behaviours, which confer competitive advantages in social interactions to top aquatic consumers. This means that the ongoing decrease n-3 LC-PUFA-rich prey (e.g. Závorka et al., 2023) is likely to have a negative impact on the capacity of fishes to maintain a high fitness in freshwater ecosystems altered by anthropogenic environmental changes.

Moreover, our data suggest that natural variation in fatty acid intake does not significantly affect brain traits such as brain mass or neuron number and density. Thus, the positive effects of high dietary n-3 LC-PUFA intake on cognitive and social competence were not linked to neural correlates traditionally associated with differences in information-processing capacity, such as increased brain size (Benson-Amram et al., 2016; Buechel et al., 2018; Sol et al., 2022), greater neuron count (Herculano-Houzel, 2017; Němec and Osten, 2020; Kverková et al., 2022) or high neuron packing density (Dicke and Roth, 2016). Instead, these effects appear to arise from biochemical changes in the brain. Social interactions are controlled by a complex network of interacting neural systems (O'Connell and Hofmann, 2011, 2012), which heavily depend on sex steroids, nonapeptides, stress modulators and reward neuromodulators, especially dopamine and serotonin (Walsh et al., 2021). Indeed, n-3 LC-PUFA have been associated with increased sensitivity of neuronal receptors (G-coupled protein receptors) to neurotransmitters, including dopamine and serotonin (Pilecky et al., 2021), while n-3 LC-PUFA deficiency leads to the degradation of dopaminergic and serotonergic neurons (Cardoso et al., 2014). Therefore, it is plausible to hypothesize that the greater social competence in individuals with higher n-3 LC-PUFA content in the brain was probably responsible for their competitive advantage (Desjardins and Fernald, 2008; Watanabe and Yamamoto, 2015). In contrast to n-3 LC-PUFA, high brain levels of n-6 PUFA can have pro-inflammatory effects (Calder, 2005), impair spatial orientation and memory, and promote anxious, less active behaviours (Nguyen et al., 2014; Delpech et al., 2015). This is in line with our finding that n-6 PUFA levels in the fish brains decreased, albeit non-significantly, the probability of individuals socially dominating the group of conspecifics.

We found that dominant individuals exhibited a faster somatic growth rate compared with the rest of the group, suggesting that the high social rank provided a fitness advantage. The positive effect of n-3 LC-PUFA on individual performance was thus closely linked to their ability to establish a dominant position within the group, probably driven by enhanced behavioural flexibility and problem-solving capacity in individuals consuming an n-3 LC-PUFA-rich diet (Mari et al., 2025). The design of our experiment and statistical model effectively controlled for the initial body size of individuals, which in juvenile salmonids positively correlates with muscular endurance (Ojanguren and Brana, 2003) and social dominance (Johnsson and Åkerman, 1998). Previous research indicates that context-relevant information such as knowledge of the contested habitat (Johnsson and Forser, 2002; Kvingedal and Einum, 2011) and awareness of the social status of the opponent, acquired through the social eavesdropping (Johnsson and Åkerman, 1998; Koene et al., 2025), can be more crucial than physical performance itself in social interactions among size-matched individuals.

We found no association between mass of the brain and telencephalon, or between fatty acid content of the brain and the somatic growth trajectories of fish during the common-garden phase prior to the stream mesocosm experiment (see Table S13). Therefore, we can confidently exclude the possibility that the growth rate of individuals during the mesocosm experiment was primarily driven by energetic reserves accumulated before the stream mesocosm experiment. Instead, the faster growth of dominant individuals appeared to be facilitated by their access to microhabitats in the mesocosms that support energy-saving behaviours (Henke-von der Malsburg et al., 2020), such as proximity to shelter and a vantage point overlooking prey-rich sections of the flume. Indeed, dominant individuals with high n-3 LC-PUFA content in their brain clearly utilized the habitat in the central part of the stream mesocosm with two shelters available within a distance of 0.5 m and with the most prey-rich habitat upstream of them, allowing them to adopt an energy-saving sit-and-wait foraging strategy (Nakano, 1995; Tunney and Steingrímsson, 2012). Interestingly, data from active RFID telemetry indicated that subdominant individuals were, on average, detected farther upstream than the dominant fish in the enclosure. This result is not intuitive, as the upstream section had the highest habitat quality in terms of substrate complexity and food availability, and our initial assumption was that the dominant fish would monopolize this section. The frequent detection of subdominant individuals at the upstream end of the enclosure was probably caused by avoidance responses to disturbance from the mobile RFID antenna (Fetherman et al., 2014). Because we always conducted telemetric screening from the downstream to the upstream end of the enclosure, subdominant individuals were probably detected farther upstream as they were unable to use the shelters already occupied by dominant fish in the central part of the flume. Subdominant individuals with a high content of n-3 LC-PUFA in the brain were able to utilize a similar microhabitat in the centre of the flume to the dominant fish but did not benefit from this position in terms of higher somatic growth, possibly because their capacity to forage was suppressed by the dominant individual (Stradmeyer et al., 2008). This finding suggests that the link between neural traits, habitat use and fitness may vary depending on the social rank of an individual (Milewski et al., 2022).

Subdominant individuals originating from the common-garden experiment with a higher n-6 PUFA content in the brain tended to occupy the downstream part of the enclosure and exhibited greater somatic growth compared with conspecifics with low n-6 PUFA content in the brain. A high n-6 PUFA content in the brain should not benefit the cognitive skills of an individual, but it is often an indicator of an increased fatty acid conversion (Pilecky et al., 2021). Therefore, this increased metabolic activity caused by fatty acid conversion in fish originating from the common-garden experiment may reflect a greater investment in somatic growth alone rather than in cognitive function itself (Colombo et al., 2023). Furthermore, overall brain size in subdominant individuals was positively correlated with somatic growth, but there was no clear effect on habitat use. These findings suggest that anatomical and biochemical neural traits may be related to the habitat use and fitness of individuals through complex behavioural and physiological effects and, in some cases, it may be challenging to tease apart causality from mere correlation between the traits. Because of the limited number of studies investigating the fitness value of cognition in natural habitats at the intra-specific level, some of these behavioural and physiological effects still remain poorly understood (Morand-Ferron et al., 2016; Logan et al., 2018). Future research mechanistically linking neurobiology, cognition and diet in wild fish and other animals should aim to establish standardized protocols for *in situ* testing of cognition (Jones et al., 2025) and should include assessment of monoamine neurochemistry (Höglund et al., 2005; Soares et al., 2018) and brain gene expression (Rosengren et al., 2018) in relation to dietary nutrients.

Our study revealed that, in wild fish, the high intake of n-3 LC-PUFA – crucial for cognitive development in juvenile trout – mainly comes from their consumption of aquatic prey, not from their trophic level. This is supported by the positive correlation between n-3 LC-PUFA content in the brain and δ^13^C values in fish muscle tissue, along with no link between n–3 LC-PUFA and δ^15^N (Bunn et al., 2013). This suggests that in freshwater ecosystems affected by human activities, changes in the length of the aquatic food web (Ward and McCann, 2017) may have less of an impact on the cognition and fitness of top consumers than shifts in the flow of dietary resources between stream and riparian habitats (Larsen et al., 2016). Our study demonstrates diet quality significantly influences brain traits, behaviour and fitness even in freshwater fish species, which otherwise display physiological adaptations for maintaining fatty acid composition optimal for brain function (Ebm et al., 2021; Závorka et al., 2021).

In conclusion, our findings indicate that insufficient dietary intake of n-3 LC-PUFA limits the ability of territorial fish to establish and maintain social dominance and optimal habitat use in natural environments. The direct link we found among n-3 LC-PUFA levels, behavioural performance and growth suggests that nutritional stress could be an overlooked mechanism affecting the population dynamics of aquatic consumers. This is particularly relevant as human impacts on freshwater ecosystems frequently lead to diminished availability of dietary n-3 LC-PUFA and other vital organic compounds through multiple pathways, including alteration of algal and invertebrate communities and reduced aquatic–terrestrial connectivity (Závorka et al., 2023; Shipley et al., 2024). Our results indicate that such changes could create a negative feedback loop where reduced n-3 LC-PUFA availability impairs the cognitive abilities that animals need to cope with environmental change. This mechanism may help explain why some populations show limited resilience to anthropogenic pressures, even when traditional metrics such as prey abundance appear sufficient (Mayntz and Toft, 2001; Shipley et al., 2024). Understanding how nutrition constrains behavioural flexibility is crucial for predicting how aquatic consumers will respond to ongoing environmental change and exposure to several simultaneous anthropogenic stressors (Jackson et al., 2021).

## Supplementary Material

10.1242/jexbio.250914_sup1 Supplementary information
