# Supplementary material for: Deficiency of vital organic nutrients in ecosystems limits brain development and fitness in wild fish
Source: J Exp Biol. 2026 Feb 12;229(3):jeb250914. doi: 10.1242/jeb.250914 (PMC12951604; doi:10.1242/jeb.250914)
Supplement: Supplementary information [file jexbio-229-250914-s1.pdf]

Supplementary Materials and Methods

Summary information on experimental fish

Table S1. *Chronology of the measurements and sample size of the experimental fish used in the stream mesocosm experiment. The last date of the mesocosms observation corresponds to the date of the final measurements of body size and the collection of tissue samples. Initial body mass and length correspond to the time of the beginning of the stream mesocosm experiment. Date of arrival indicates when the individual arrived at the husbandry facility at WasserCluster Lunz. cg = common garden * = values based only on data from 02/08/2023 to 06/08/2023*

Origin	*Sampling site coordinates*	*Date of arrival*	*Dates of mesocosm observation*	*Water temperature [ºC] mean±SD*	*Experimental dietary treatement*	*Sample size (males/females/NA)*	*Initial body mass [g] mean±SD*	*Initial fork lenght [mm] mean±SD*
Attersee - hatchery/cg	-	04.11.22	16.05. - 25.05. 2023	10.3 ± 1.5	n3+	3/3/3	32.0 ± 10.4	143±13
					n3-	8/7/0	25.5 ± 9.1	133±15
Drau-hatchery/cg	-	31.10.22	26.05. - 04.06. 2023	10.1 ± 2.3	n3+	3/9/0	34.5±12.2	147±15
					n3-	7/5/0	31.7±6.4	142±9
Kamp - hatchery/cg	-	31.10.22	06.06. - 15.06.2023	8.4 ± 0.6	n3+	7/5/0	14.2±4.2	110±9
					n3-	8/4/0	13.1±6.4	106±14
Kleiner Kamp - wild	48.4415322N, 15.0118947E	29.05.23	16.06. - 25.06.2023	8.8 ± 0.8	natural diet	10/14/0	32.5±8.4	149±13
Lohnbach - wild	48.4784950N, 15.0216714E	13.06.23	27.06 - 06.07.2023	9.3 ± 0.6	natural diet	9/15/0	17.5±5.6	120±12
Ybbs - wild/cg	47.8627883N, 15.0405114E	24.10.22	18.07. - 27.07. 2023	NA	n3+	6/5/0	16.9±4.8	115±10
					n3-	5/8/0	16.9±8.9	112±16
Weissensee – hatchery/cg	-	31.10.22	28.07. - 06.08. 2023	10.9 ± 0.7*	n3+	7/4/0	21.6±6.3	122±20
					n3-	8/2/0	33.1±23.1	134±27
Ybbs - wild	47.8627883N, 15.0405114E	03.08.23	08.08. - 17.08.2023	11.9 ± 0.5	natural diet	12/7/5	23.9±7.0	133±13
Salza – wild	47.7663600N, 14.8481864E	25.07.23	18.08. - 27.08.2023	12.2 ± 0.6	natural diet	6/15/3	15.8±2.7	117±7

Journal of Experimental Biology • Supplementary information

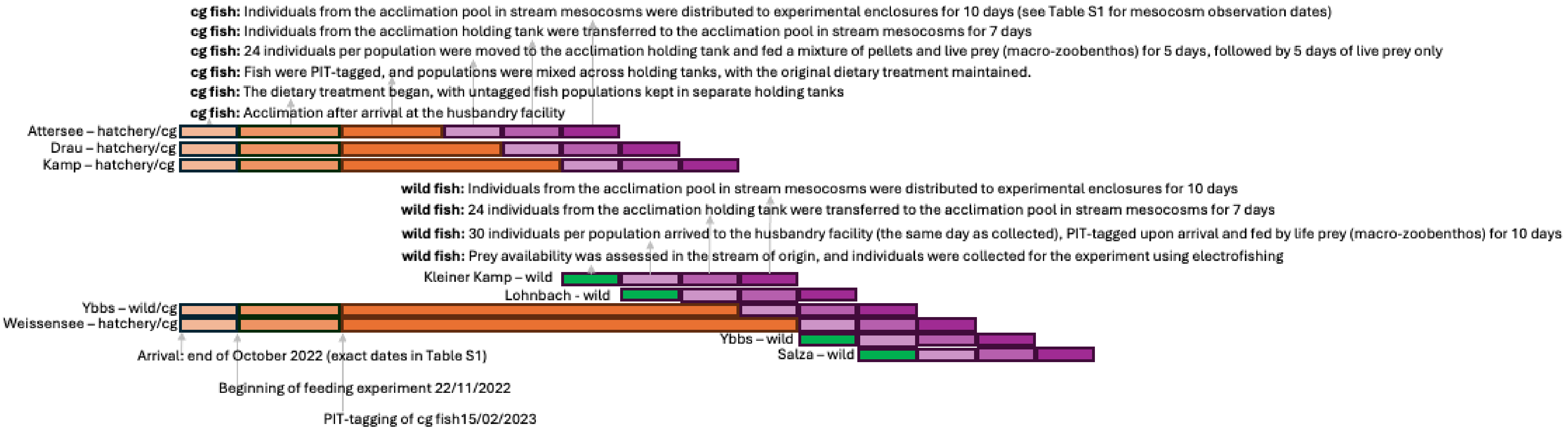

Fig. S1. Timeline design of the entire experiment. Major experimental periods for cg-fish and wild fish are indicated. The length of bars represents the relative duration of each experimental period. Exact dates common to all populations are indicated below the diagram, while population-specific dates are listed in Table S1.

Journal of Experimental Biology • Supplementary information

Diet Composition for the Common Garden Experiment

Table S2. *Composition of the experimental diet as indicated by the producer GARANT™, Austria*

Feed components	High n-3 LC-PUFA	Low n-3 LC-PUFA
Fish meal, Super prime, 67 % XP	20	-
Sunflower protein concentrate, 45 % XP	10	10
Blood meal SD	7	7
Hemoglobin powder	3	3
Poultry meal	14.8	20
Wheat gluten, 80 % XP	2	8.5
Soy protein concentrate	-	2.9
Soybean meal HP	-	5
Wheat, feed quality	13.4	10.9
Wheat feed flower	10	10
Fish oil	7.9	-
Rapeseed oil	10.9	14.8
Lin oil	-	5
Monocalciumphosphate	-	1,1
Lysine-HCl	0.06	0.69
Methionine	0.22	0.40
Threonine	0.09	0.24
Premix	0,6	0.6
Digestible energy, MJ	20.3	20.3
Crude protein, %	42	42
Crude fat, %	23	22
Crude fibre, %	1.4	1.5
P, %	1.06	1.05
Lysine, %	2.65	2.65
Methionine, %	1.0	1.0
Met + Cys, %	1.45	1.5
Threonine, %	1.6	1.6

Table S3. *Average concentration of fatty acids in experimental diets (N = 3 for each diet). Note that we report only the fatty acids that have been detected in concentrations higher than 1%. Data from Závorka et al. (2021)*

	High n-3 LC-PUFA		Low n-3 LC-PUFA	
**Common name**	µg / mg dry weight	%	µg / mg dry weight	%
Palmitic acid (C16:0)	27.2	13.70	19.5	8.97
Stearic acid (C18:0)	6.9	3.45	5.7	2.64
7-Hexadecanoic acid (C16:1n-9)	0.2	0.11	0.4	0.22
Palmitoleic acid (C16:1n-7)	2.9	1.31	7.1	3.56
Oleic acid (C18:1n-9)	69.2	34.78	118.6	54.58
Vaccenic acid (C18:1n-7)	4.3	2.15	4.3	1.99
Linoleic acid (C18:2n-6)	31.4	15.77	40.0	18.38
Gondonic acid (C20:1n-9)	3.3	1.49	7.2	3.62
Alpha-Linolenic acid (C18:3n-3)	9.6	4.81	11.5	5.25
Dihomo-g-linolenic acid (C20:3n-6)	0.1	0.04	0.3	0.17
Arachidonic acid (C20:4n-6)	0.8	0.38	0.3	0.14
Eicosapentaenoic acid (C20:5n-3)	7.3	3.69	2.0	0.90
Nervonic acid (C24:1n-9)	0.7	0.32	1.1	0.54
Docosatetraenoic acid (C22:4n-6)	0.0	0.00	0.0	0.00
Docosahexaenoic acid (C22:6n-3)	9.8	4.95	2.0	0.89
Sum of n-3 LC-PUFA	17.1	8.64	4.0	1.79
Sum of n-6 PUFA	32.3	16.19	40.60	18.69
Sum of the remaining fatty acids	6.4	2.98	16.4	8.24

References

Závorka, L., Crespel, A., Dawson, N. J., Papatheodoulou, M., Killen, S. S., & Kainz, M. J. (2021). Climate change-induced deprivation of dietary essential fatty acids can reduce growth and mitochondrial efficiency of wild juvenile salmon. *Functional Ecology*, *35*(9), 1960-1971.

Further description of the stream mesocosms

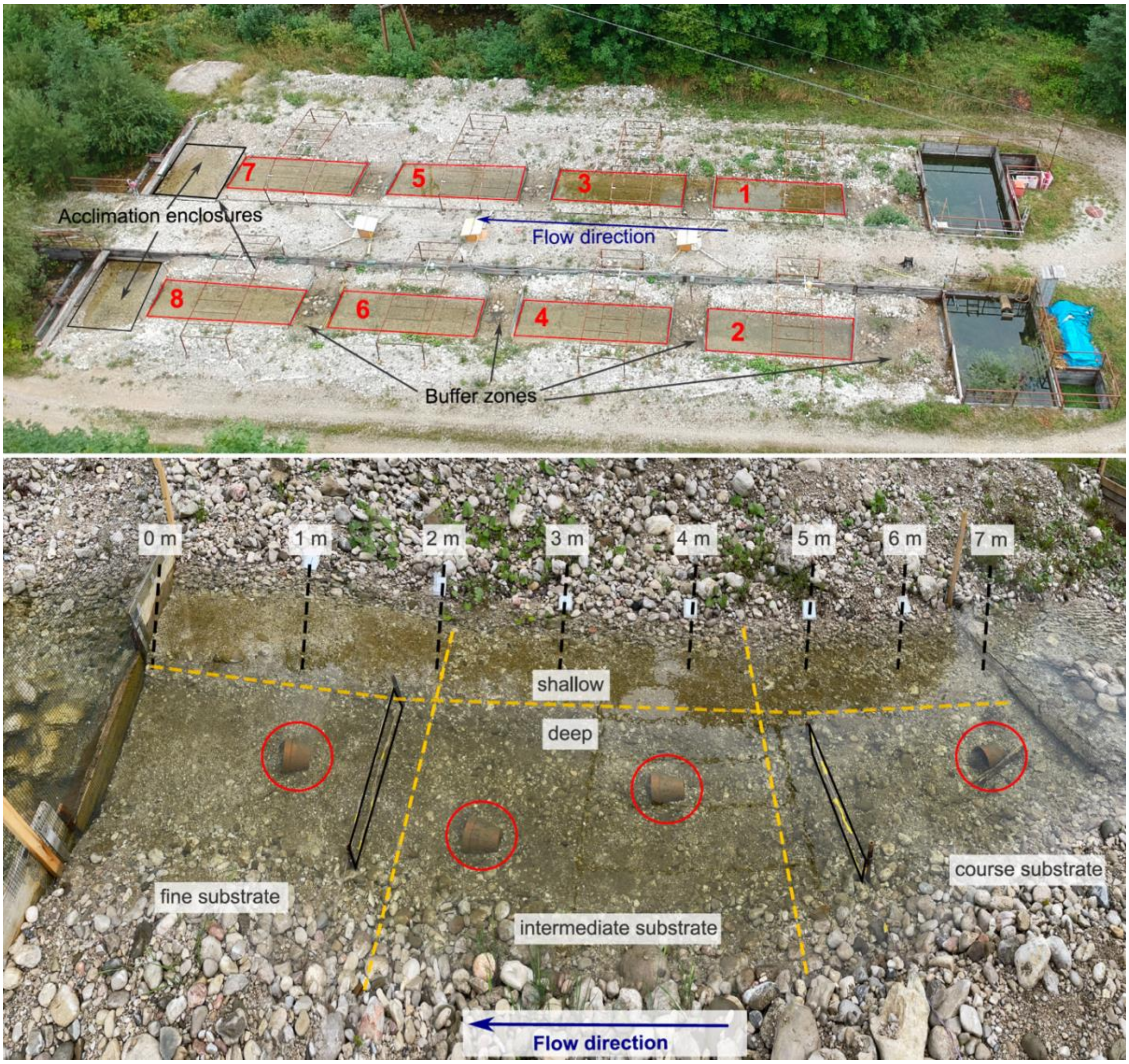

Fig. S2. Overview of the stream mesocosms. Top: An aerial view of the flumes showing the distribution of experimental enclosures (red rectangles, numbered 1–8) separated by buffer zones, and acclimation enclosures (two black rectangles on the left). The metallic frame above each section supported cameras, while booths along the central path between the two flumes housed the control units for stationary RFID telemetry, video recording, and temperature loggers. **Bottom:** A detailed view of an experimental enclosure showing the arrangement of shelters (red circles), RFID antennas (black rectangles), and various habitats categorized by water depth and substrate grain size.

Journal of Experimental Biology • Supplementary information

Telemetry and video-surveillance

Active telemetry in the stream mesocosms was conducted every morning between 9:00 and 11:00. Experimental enclosures were scanned from downstream to upstream using a portable antenna three times daily, with a 30-minute pause between individual scanning sessions. When detected, the longitudinal position (to the nearest 0.5 m) and the individual's ID were recorded in a protocol. Additionally, sets of two custom-made, flow-through rectangular stationary RFID antennas (25 × 100 cm) were installed transversely in each experimental enclosure at longitudinal positions of 2 and 5 meters (see Fig. S3.1). The stationary antennas covered only the deep section of the channel. The total of 16 stationary antennas were connected to 4 readers (each reader collecting data from 4 antennas). To avoid interference between antennas and maintain high detection range in the whole diameter of antennas, we were able to run only 8 antennas (i.e., pairs in four experimental enclosures) at time. Therefore, enclosures 1 to 4 had stationary antennas active on even days and enclosures 5 to 8 on odd days of the observation. All stationary antennas were turned off every day between 9.00 and 11.30 to prevent interference with the portable RFID antenna during active telemetry. The detection range of the stationary antennas was checked every morning and data from readers were downloaded every 5 days. Each enclosure was equipped with a camera capable of day and night vision recording connected by an ethernet cable to a recording system (RLN16-410, Reolink, China). For recording in nighttime each enclosure was illuminated by two infra-red spotlights. Spotlights and cameras were placed on a metal frame above the enclosure (Fig. S3.1). Cameras in each enclosure were at 2.90 m above the bottom of the channel at a longitudinal position 4.5 m from the downstream end of the enclosure, which allowed the camera frame to cover the whole area of the experimental enclosure with exception of < 0.5 m of the downstream end (see Fig S3.3). Cameras were activated each day in ten 30 minutes intervals starting at 01:00, 03:00, 05:00, 07:00, 13:00, 15:00, 17:00, 19:00, 21:00, and 23:00.

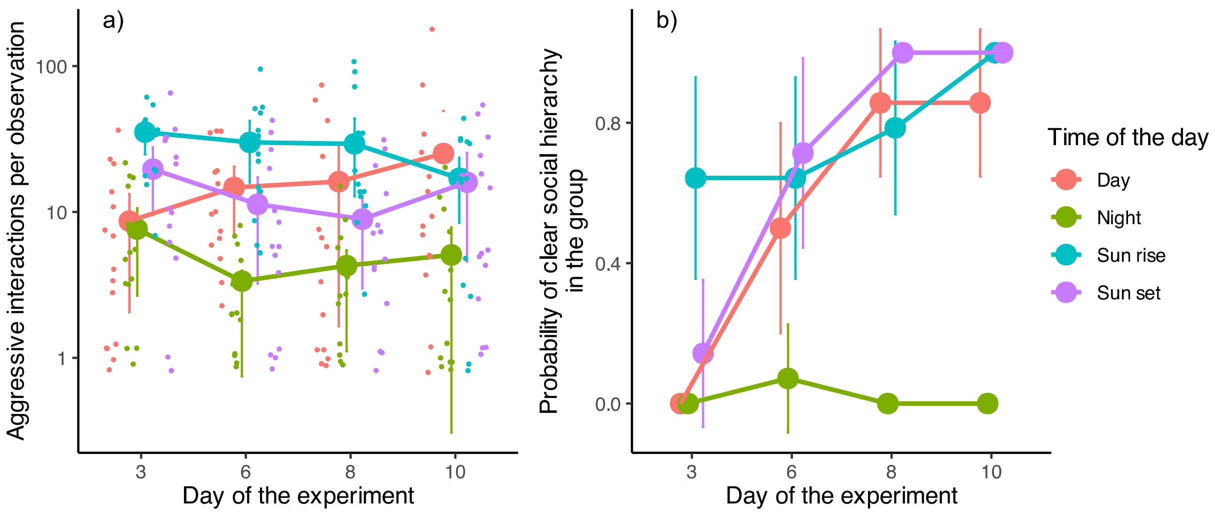

Fig. S3. Error bar plot (mean ± 95% CI) showing (a) the number of aggressive interactions per observation and (b) the probability of identifying a clear social hierarchy during a given observation. The data are derived from ethological observations of a subset of video recordings collected in a stream mesocosm during four experimental rounds with fish from wild populations. Each observation lasted 30 minutes, and recordings were selected from paired days: 2/3, 5/6, 7/8, and 9/10 of the experiment. Observations were conducted at 15:00 (Day) and 21:00 (Sunset) on the first day of each pair, and at 01:00 (Night) and 05:00 (Sunrise) on the second day. The video recordings allowed for assessment of aggressive interactions within the entire group but did not differentiate between the aggressiveness of individual fish. Social dominance was scored as “1” when a clearly dominant individual was present and as “0” otherwise.

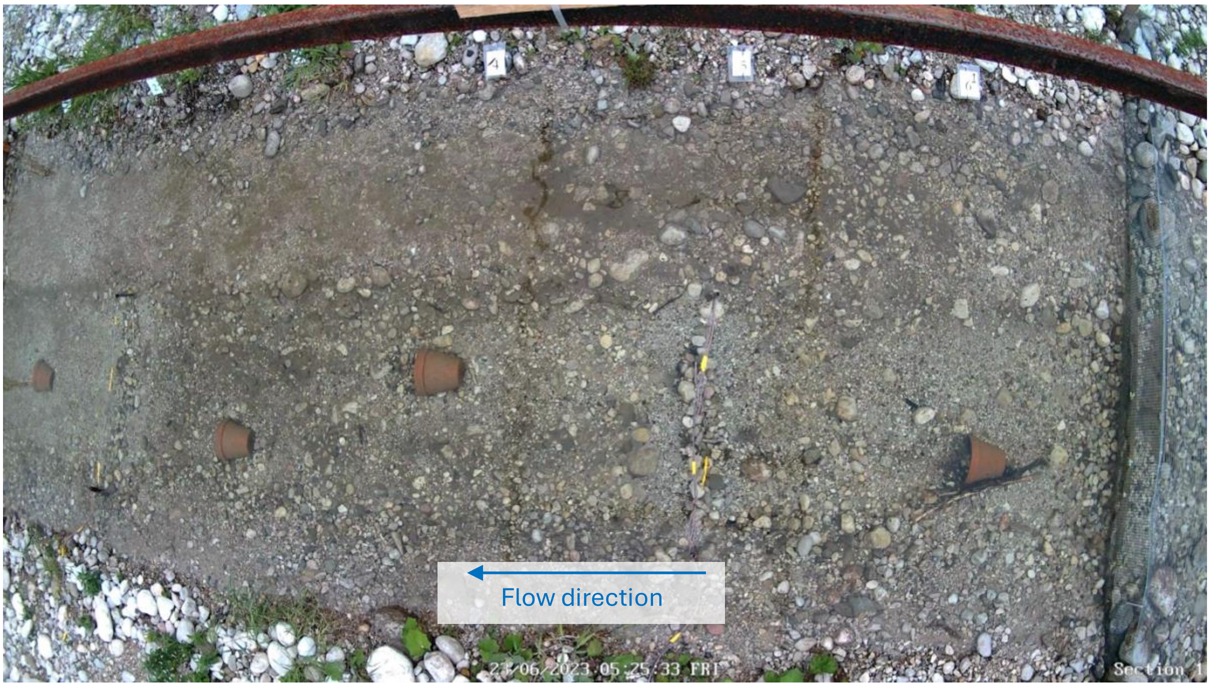

Fig. S4. View of the camera in the enclosure of the stream mesocosm

Journal of Experimental Biology • Supplementary information

Table S4. Taxonomic composition of the most common taxa of benthic macro-invertebrates collected in Seebach and used for inoculation of the mesocosms flumes. The list is ordered from the most to the least common taxa in the sample.

**Taxa**
Hydropsyche sp.
Sericostoma sp.
Micrasema minimum
Glossosoma sp./*Agapetus* sp.
Epeorus sp.
Ecdyonurus sp.
Baetis sp.
Rhithrogena sp.
Protonemura sp.
Amphinemura sp.
Habroleptoides sp.
Gammarus fossarum
Atherix ibis
Tabanus sp.
Tipulidae Gen. sp.
Ancylus fluviatilis

Journal of Experimental Biology • Supplementary information

Effect of diet on neural traits

Table S5. Full model report on the effect of diet quality on fatty acid composition and overall brain mass for fish from the common garden experiment. At the population level, effects are calculated relative to fish from the Kamp River.

**Brain n-3 LC-PUFA ~**	*Estimate*	*s.e.*	*t-value*	*Pr(>|t|)*
Diet n3-	-1.823	0.444	-4.108	<0.001
Population Attersee	-0.734	0.805	-0.912	0.364
Population Ybbs	-1.592	0.767	-2.076	0.040
Population Drau	1.427	0.764	1.868	0.064
populationWeissensee	-1.692	0.784	-2.159	0.033
Sex Male	0.621	0.447	1.388	0.168
Fork length	-0.039	0.013	-2.875	0.005
**Brain n-6 PUFA ~**				
Diet n3-	1.031	0.106	9.721	<0.001
Population Attersee	-0.334	0.192	-1.733	0.086
Population Ybbs	-0.018	0.183	-0.098	0.923
Population Drau	-0.457	0.183	-2.503	0.014
populationWeissensee	0.462	0.187	2.463	0.015
Sex Male	0.041	0.107	0.382	0.703
Fork length	0.009	0.003	2.816	0.006
**Brain MUFA ~**				
Diet n3-	0.919	0.455	2.020	0.046
Population Attersee	1.258	0.825	1.525	0.130
Population Ybbs	3.287	0.786	4.183	<0.001
Population Drau	-0.203	0.783	-0.259	0.796
populationWeissensee	3.316	0.803	4.127	<0.001
Sex Male	-0.544	0.458	-1.188	0.237
Fork length	0.020	0.014	1.484	0.140
**Brain SFA ~**				
Diet n3-	-0.176	0.211	-0.832	0.407
Population Attersee	-0.244	0.383	-0.638	0.525
Population Ybbs	-1.731	0.365	-4.745	<0.001
Population Drau	-0.478	0.363	-1.316	0.191
populationWeissensee	-2.130	0.373	-5.711	<0.0001
Sex Male	-0.085	0.213	-0.398	0.691
Fork length	0.006	0.006	0.990	0.324
**Brain mass ~**				
Diet n3-	2.162	1.964	1.101	0.273
Population Attersee	-12.766	3.532	-3.614	<0.001
Population Ybbs	-2.016	3.381	-0.596	0.552
Population Drau	-6.405	3.395	-1.886	0.062
populationWeissensee	-1.708	3.486	-0.490	0.625
Sex Male	-0.344	1.994	-0.173	0.863
Fork length	1.160	0.061	19.147	<0.001

Journal of Experimental Biology • Supplementary information

Table S6. Full model report on the effect of diet quality on fatty acid composition and overall brain mass for wild fish. At the population level, effects are calculated relative to fish from the Kleiner Kamp River.

**Brain n-3 LC-PUFA ~**	*Estimate*	*s.e.*	*t-value*	*Pr(>|t|)*
δ13C	-9.101	2.084	-4.367	<0.001
δ15N	-1.384	1.501	-0.922	0.359
Population Lohnbach	0.157	0.649	0.241	0.810
Population Salza	-2.003	0.769	-2.603	0.011
Population Ybbs	-2.978	0.848	-3.510	0.001
Sex Male	-0.227	0.361	-0.628	0.531
Fork length	-0.031	0.012	-2.552	0.012
**Brain n-6 PUFA ~**				
δ13C	1.835	0.606	3.029	0.003
δ15N	0.439	0.438	1.004	0.318
Population Lohnbach	0.212	0.187	1.134	0.259
Population Salza	-0.773	0.224	-3.450	0.001
Population Ybbs	-1.303	0.247	-5.267	<0.001
Sex Male	0.053	0.105	0.507	0.614
Fork length	0.009	0.003	2.528	0.013
**Brain MUFA ~**				
δ13C	8.571	2.524	3.396	0.001
δ15N	1.096	1.824	0.601	0.549
Population Lohnbach	-0.256	0.781	-0.328	0.744
Population Salza	3.249	0.933	3.481	0.001
Population Ybbs	4.658	1.031	4.518	<0.001
Sex Male	0.427	0.436	0.979	0.330
Fork length	0.037	0.014	2.563	0.012
**Brain SFA ~**				
δ13C	-2.325	1.024	-2.271	0.025
δ15N	0.564	0.740	0.762	0.448
Population Lohnbach	-0.208	0.317	-0.656	0.513
Population Salza	-0.334	0.379	-0.883	0.380
Population Ybbs	-0.723	0.418	-1.729	0.087
Sex Male	-0.120	0.177	-0.680	0.498
Fork length	-0.024	0.006	-4.073	<0.001
**Brain mass ~**				
δ13C	24.285	15.436	1.573	0.118
δ15N	-2.490	11.169	-0.223	0.824
Population Lohnbach	-1.850	4.634	-0.399	0.690
Population Salza	-6.367	5.572	-1.143	0.256
Population Ybbs	-2.648	6.105	-0.434	0.665
Sex Male	-2.591	2.623	-0.988	0.325
Fork length	1.602	0.081	19.822	<0.001

Journal of Experimental Biology • Supplementary information

Table S7. Full model report on the effect of diet quality on mass of brain regions and total number of neurons in brain for fish from the common garden experiment. At the population level, effects are calculated relative to fish from the Kamp River.

**Tel mass ~**	*Estimate*	*s.e.*	*t-value*	*Pr(>|t|)*
Diet n3-	0.112	0.134	0.835	0.405
Population Attersee	-0.385	0.253	-1.523	0.130
Population Ybbs	0.241	0.231	1.043	0.299
Population Drau	0.180	0.235	0.768	0.444
populationWeissensee	-0.315	0.238	-1.327	0.187
Sex Male	0.139	0.136	1.020	0.310
Fork length	-0.025	0.008	-3.007	0.003
Brain mass	0.073	0.006	12.028	<0.001
**OT mass ~**				
Diet n3-	0.899	0.481	1.868	0.064
Population Attersee	0.991	0.905	1.094	0.276
Population Ybbs	-1.100	0.826	-1.332	0.185
Population Drau	-0.377	0.840	-0.449	0.654
populationWeissensee	0.946	0.851	1.112	0.268
Sex Male	-0.619	0.486	-1.272	0.206
Fork length	0.010	0.029	0.327	0.744
Brain mass	0.257	0.022	11.771	<0.001
**Cb mass ~**				
Diet n3-	0.177	0.325	0.546	0.586
Population Attersee	1.335	0.612	2.183	0.031
Population Ybbs	0.596	0.558	1.068	0.288
Population Drau	0.601	0.567	1.060	0.291
populationWeissensee	0.476	0.575	0.828	0.409
Sex Male	0.046	0.329	0.139	0.890
Fork length	-0.006	0.020	-0.319	0.750
Brain mass	0.234	0.015	15.853	<0.001
**RoB mass ~**				
Diet n3-	-1.123	0.510	-2.204	0.029
Population Attersee	-1.999	0.958	-2.086	0.039
Population Ybbs	0.398	0.874	0.455	0.650
Population Drau	-0.368	0.889	-0.414	0.679
populationWeissensee	-0.834	0.901	-0.926	0.356
Sex Male	0.487	0.515	0.946	0.346
Fork length	0.020	0.031	0.629	0.531
Brain mass	0.418	0.023	18.103	<0.001
**Brain neuron N° ~**				
Diet n3-	1681721	1593396	1.055	0.295
Population Attersee	-2046709	2663894	-0.768	0.445
Population Ybbs	-2520612	2776232	-0.908	0.367
Population Drau	-1812258	2640266	-0.686	0.495
populationWeissensee	-283	1599428	0.000	1.000
Sex Male	143728	101831	1.411	0.163
Fork length	56869	71613	0.794	0.430
Brain mass	1681721	1593396	1.055	0.295

Journal of Experimental Biology • Supplementary information

Table S8. Full model report on the effect of diet quality on mass of brain regions and total number of neurons for wild fish. At the population level, effects are calculated relative to fish from the Kleiner Kamp River.

**Tel mass ~**	*Estimate*	*s.e.*	*t-value*	*Pr(>|t|)*
δ13C	-0.829	1.128	-0.735	0.464
δ15N	0.574	0.808	0.711	0.479
Population Lohnbach	0.329	0.335	0.981	0.329
Population Salza	0.196	0.405	0.484	0.630
Population Ybbs	0.048	0.442	0.108	0.914
Sex Male	-0.021	0.190	-0.110	0.913
Fork length	-0.013	0.012	-1.076	0.284
Brain mass	0.078	0.007	11.401	<0.001
**OT mass ~**				
δ13C	4.253	4.145	1.026	0.307
δ15N	-1.044	2.967	-0.352	0.726
Population Lohnbach	-2.701	1.232	-2.193	0.030
Population Salza	0.068	1.488	0.046	0.964
Population Ybbs	4.104	1.623	2.529	0.013
Sex Male	-0.297	0.700	-0.424	0.672
Fork length	-0.037	0.046	-0.805	0.423
Brain mass	0.251	0.025	10.015	<0.001
**Cb mass ~**				
δ13C	-3.204	2.145	-1.493	0.138
δ15N	-0.541	1.536	-0.352	0.725
Population Lohnbach	-0.263	0.638	-0.413	0.680
Population Salza	-0.858	0.770	-1.114	0.268
Population Ybbs	-0.790	0.840	-0.941	0.349
Sex Male	-0.090	0.362	-0.248	0.804
Fork length	0.101	0.024	4.290	<0.001
Brain mass	0.169	0.013	13.007	<0.001
**RoB mass ~**				
δ13C	-0.007	4.759	-0.002	0.999
δ15N	0.366	3.407	0.107	0.915
Population Lohnbach	2.380	1.414	1.682	0.095
Population Salza	0.160	1.709	0.094	0.926
Population Ybbs	-3.533	1.863	-1.896	0.061
Sex Male	0.336	0.803	0.418	0.677
Fork length	-0.073	0.052	-1.393	0.167
Brain mass	0.489	0.029	16.972	<0.001
**Brain neuron N° ~**				
δ13C	-6883708	13173976	-0.523	0.605
δ15N	6978477	6603137	1.057	0.299
Population Lohnbach	-4395093	3408509	-1.289	0.207
Population Salza	808130	4594949	0.176	0.862
Population Ybbs	-1619504	4635959	-0.349	0.729
Sex Male	-2356283	1877626	-1.255	0.219
Fork length	304964	157895	1.931	0.063
Brain mass	-85049	80972	-1.050	0.302

Journal of Experimental Biology • Supplementary information

Table S9. Full model report on the effect of diet quality on number of neurons in brain regions for fish from the common garden experiment. At the population level, effects are calculated relative to fish from the Kamp River.

**Tel neuron N°~**	*Estimate*	*s.e.*	*t-value*	*Pr(>|t|)*
Diet n3-	-12932	42796	-0.302	0.764
Population Attersee	-108130	73196	-1.477	0.145
Population Drau	1342	74651	0.018	0.986
populationWeissensee	25466	74576	0.341	0.734
Sex Male	54203	43436	1.248	0.217
Tel mass	-14310	21479	-0.666	0.508
Fork length	3427	1924	1.781	0.080
**OT neuron N°~**				
Diet n3-	210645	244554	0.861	0.392
Population Attersee	94953	386953	0.245	0.807
Population Drau	-312647	423165	-0.739	0.463
populationWeissensee	-242181	399908	-0.606	0.547
Sex Male	39019	244397	0.160	0.874
OT mass	56307	31299	1.799	0.077
Fork length	8097	12796	0.633	0.529
**Cb neuron N°~**				
Diet n3-	1388708	1376554	1.009	0.317
Population Attersee	-1723613	2177138	-0.792	0.432
Population Drau	-1752774	2417110	-0.725	0.471
populationWeissensee	-1324466	2273647	-0.583	0.562
Sex Male	-77868	1397594	-0.056	0.956
Cb mass	241071	240164	1.004	0.319
Fork length	109163	82313	1.326	0.190
**RoB neuron N°~**				
Diet n3-	-116133	91809	-1.265	0.211
Population Attersee	-164275	157392	-1.044	0.301
Population Drau	-137928	161781	-0.853	0.397
populationWeissensee	-87774	152969	-0.574	0.568
Sex Male	29723	92293	0.322	0.748
RoB mass	27130	8184	3.315	0.002
Fork length	-900	5352	-0.168	0.867

Journal of Experimental Biology • Supplementary information

Table S10. Full model report on the effect of diet quality on number of neurons in brain regions for wild fish. At the population level, effects are calculated relative to fish from the Kleiner Kamp River.

**Tel neuron N°~**	*Estimate*	*s.e.*	*t-value*	*Pr(>|t|)*
δ13C	535546	443496	1.208	0.236
δ15N	-88870	223375	-0.398	0.693
Population Lohnbach	71459	115187	0.620	0.540
Population Salza	235486	154910	1.520	0.139
Population Ybbs	320536	160649	1.995	0.055
Sex Male	-132051	64569	-2.045	0.049
Tel mass	47337	23237	2.037	0.050
Fork length	-7490	4076	-1.838	0.076
**OT neuron N°~**				
δ13C	2736218	1951558	1.402	0.171
δ15N	634616	933948	0.679	0.502
Population Lohnbach	-5611	492346	-0.011	0.991
Population Salza	748875	682091	1.098	0.281
Population Ybbs	1301267	700636	1.857	0.073
Sex Male	103068	268466	0.384	0.704
OT mass	124517	53760	2.316	0.027
Fork length	-43356	22956	-1.889	0.068
**Cb neuron N°~**				
δ13C	-9032669	12657129	-0.714	0.481
δ15N	5456493	6057265	0.901	0.375
Population Lohnbach	-3791957	3193188	-1.188	0.244
Population Salza	1952065	4423809	0.441	0.662
Population Ybbs	-902044	4544086	-0.199	0.844
Sex Male	-1649238	1741178	-0.947	0.351
Cb mass	200687	348669	0.576	0.569
Fork length	102899	148886	0.691	0.495
**RoB neuron N°~**				
δ13C	855198	776115	1.102	0.279
δ15N	510888	379097	1.348	0.188
Population Lohnbach	106166	196169	0.541	0.592
Population Salza	403951	259614	1.556	0.130
Population Ybbs	227096	263060	0.863	0.395
Sex Male	-234128	107752	-2.173	0.038
RoB mass	25164	8605	2.924	0.006
Fork length	-12714	8092	-1.571	0.126

Journal of Experimental Biology • Supplementary information

Dominance and SGR

Table S11. Full model report on the effect of brain quality on the probability of attaining social dominance within a group of conspecifics, including all fish. Random effect of population was not included in the models because it explained variance = 0.

**Dominance~**	*Estimate*	*s.e.*	*z-value*	*Pr(>|z|)*
Brain n-3 LC-PUFA Rank	-0.458	0.185	-2.470	0.013
Origin Wild	0.216	0.499	0.432	0.665
Sex Male	0.476	0.500	0.953	0.340
Body size Rank	-0.653	0.191	-3.415	<0.001
Brain n-6 PUFA Rank	0.369	0.161	2.29	0.022
Origin	0.263	0.494	0.533	0.594
Sex Male	0.520	0.495	1.049	0.294
Body size Rank	-0.608	0.187	-3.248	0.001
Brain mass Rank	-0.124	0.166	-0.747	0.455
Origin	-0.052	0.455	-0.115	0.908
Sex Male	0.660	0.457	1.443	0.149
Body size Rank	-0.511	0.173	-2.947	0.003
Tel mass Rank	-0.072	0.150	-0.479	0.632
Origin	-0.072	0.454	-0.159	0.874
Sex Male	0.711	0.459	1.549	0.121
Body size Rank	-0.567	0.160	-3.353	<0.001

Journal of Experimental Biology • Supplementary information

Table S12. Full model report on the effect of social dominance on body mass growth rate for all individuals in stream mesocosms, and the effect of brain quality on body mass growth rate for subdominant individuals.

**SGR mass~**	*Estimate*	*s.e.*		*t-value*	p-value
Dominance 0	0.243	0.168		2.556	0.011
Origin Wild	-0.153	0.218		-0.701	0.484
Sex Male	0.078	0.070		1.107	0.268
Body size Rank	0.007	0.021		0.310	0.756
Random effects	*Variance*	*s.d.*	*Num. of obs.*	*Num. of groups*	
population	*0.093*	*0.304*	*174*	*9*	
Brain n-3 PUFA Rank	-0.024	0.028		-0.853	0.393
Origin Wild	-0.098	0.234		-0.418	0.676
Sex Male	0.154	0.088		1.747	0.081
Body size Rank	0.003	0.028		0.125	0.900
Random effects	*Variance*	*s.d.*	*Num. of obs.*	*Num. of groups*	
population	*0.100*	*0.318*	*119*	*9*	
Brain n-6 PUFA Rank	-0.130	0.037		-3.465	0.001
Origin Wild	-0.533	0.276		-1.928	0.054
Sex Male	0.118	0.086		1.387	0.165
Body size Rank	-0.000	0.026		-0.009	0.992
Brain n-6 PUFA Rank : Origin Wild	0.145	0.052		2.759	0.006
Random effects	*Variance*	*s.d.*	*Num. of obs.*	*Num. of groups*	
population	*0.093*	*0.305*	*119*	*9*	
Brain mass Rank	-0.079	0.027		-2.963	0.003
Origin Wild	-0.079	0.222		-0.360	0.719
Sex Male	0.200	0.083		2.404	0.016
Body size Rank	0.035	0.026		1.337	0.181
Random effects	*Variance*	*s.d.*	*Num. of obs.*	*Num. of groups*	
population	*0.090*	*0.301*	*122*	*9*	
Tel mass Rank	-0.037	0.025		-1.437	0.151
Origin Wild	-0.081	0.224		-0.362	0.718
Sex Male	0.188	0.086		2.190	0.029
Body size Rank	0.010	0.025		0.412	0.680
Random effects	*Variance*	*s.d.*	*Num. of obs.*	*Num. of groups*	
population	*0.091*	*0.302*	*122*	*9*	

Journal of Experimental Biology • Supplementary information

Table S13. Full model report on the effects of brain quality on body mass growth rate during the common garden experiment preceding the stream mesocosm test. The measurements of brain quality and size rank are the same as used in the models from the stream mesocosm.

**SGR mass~**	*Estimate*	*s.e.*	*t-value*		p-value
Brain n-3 PUFA Rank	-0.011	0.012	-0.912		0.362
Sex Male	0.008	0.035	0.243		0.808
Body size Rank	-0.030	0.011	-2.732		0.006
Random effects	*Variance*	*s.d.*	*Num. of obs.*	*Num. of groups*	
population	*0.033*	*0.012*	*124*	*5*	
Brain n-6 PUFA Rank	0.011	0.012	0.924		0.355
Sex Male	0.013	0.035	0.382		0.703
Body size Rank	-0.031	0.011	-2.842		0.004
Random effects	*Variance*	*s.d.*	*Num. of obs.*	*Num. of groups*	
population	*0.029*	*0.169*	*124*	*5*	
Brain mass Rank	-0.007	0.014	-0.486		0.627
Sex Male	0.004	0.034	0.124		0.902
Body size Rank	-0.026	0.013	-2.026		0.043
Random effects	*Variance*	*s.d.*	*Num. of obs.*	*Num. of groups*	
population	*0.032*	*0.178*	*128*	*5*	
Tel mass Rank	-0.001	0.012	-0.078		0.938
Sex Male	0.004	0.034	0.116		0.908
Body size Rank	-0.029	0.011	-2.722		0.006
Random effects	*Variance*	*s.d.*	*Num. of obs.*	*Num. of groups*	
population	*0.031*	*0.177*	*128*	*5*	

Journal of Experimental Biology • Supplementary information

Position of individuals in the stream mesocosm

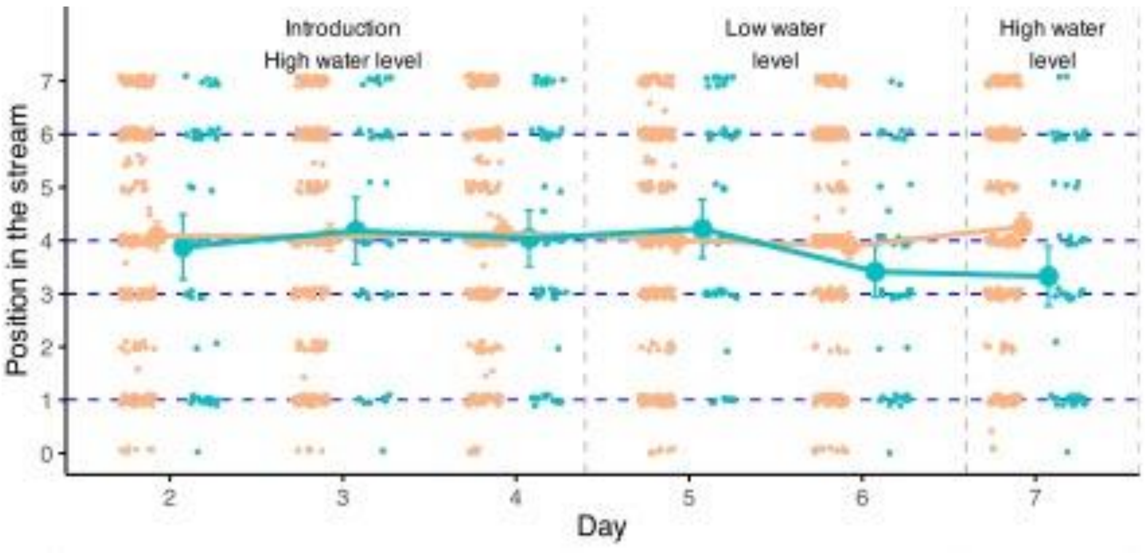

Figure S5. Error bar plot (mean ± 95% CI) showing distribution of positions of dominant (green) and subdominant (orange) individuals across all experimental rounds. Position in the stream indicates longitudinal distance from the downstream end of each enclosure in meters. Horizontal dashed lines indicate position of shelters in the enclosure, vertical dashed lines indicate limits between periods with high and low water level.

Journal of Experimental Biology • Supplementary information

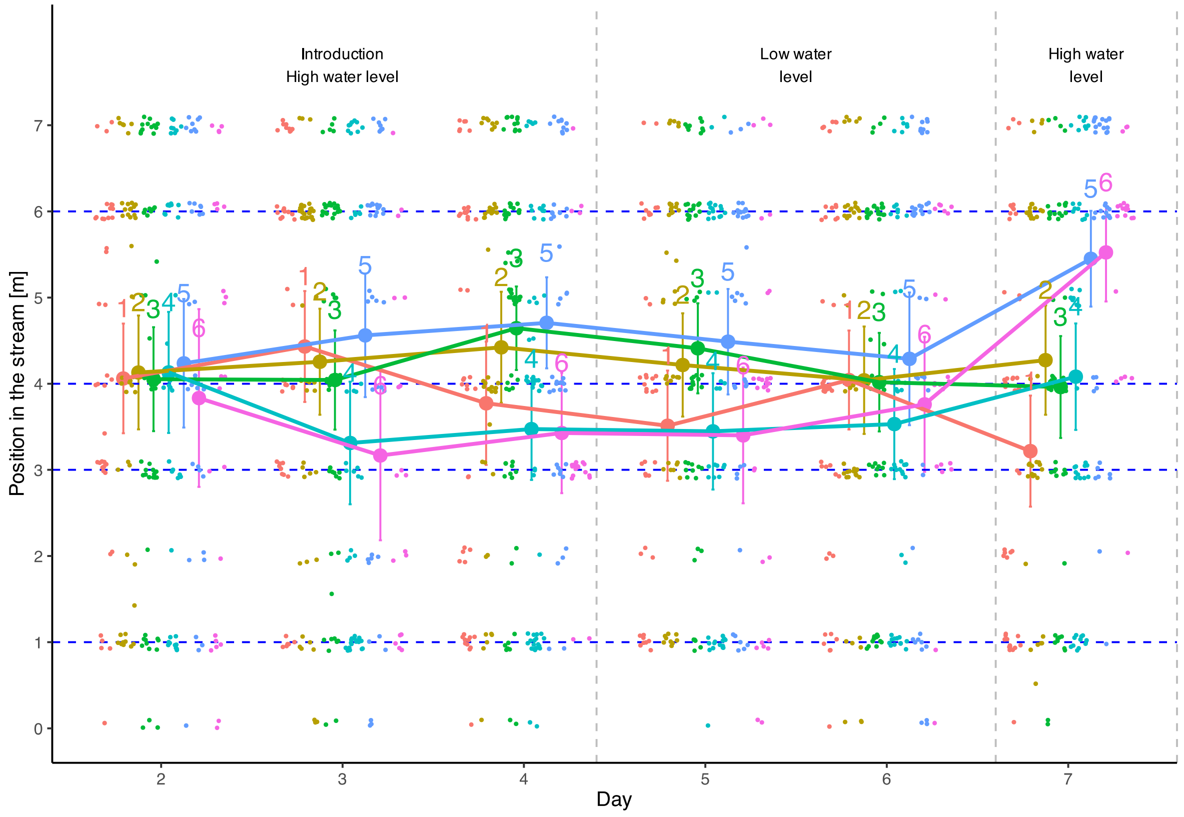

Figure S6. Error bar plot (mean ± 95% CI) showing the distribution of positions of subdominant individuals across all experimental rounds in relation to their n-3 PUFA rank (1–6) indicated by the number above the circle. Position in the stream indicates the longitudinal distance from the downstream end of each enclosure, measured in meters. Horizontal dashed lines represent the position of shelters in the enclosure, while vertical dashed lines indicate the boundaries between periods of high and low water levels.

Journal of Experimental Biology • Supplementary information

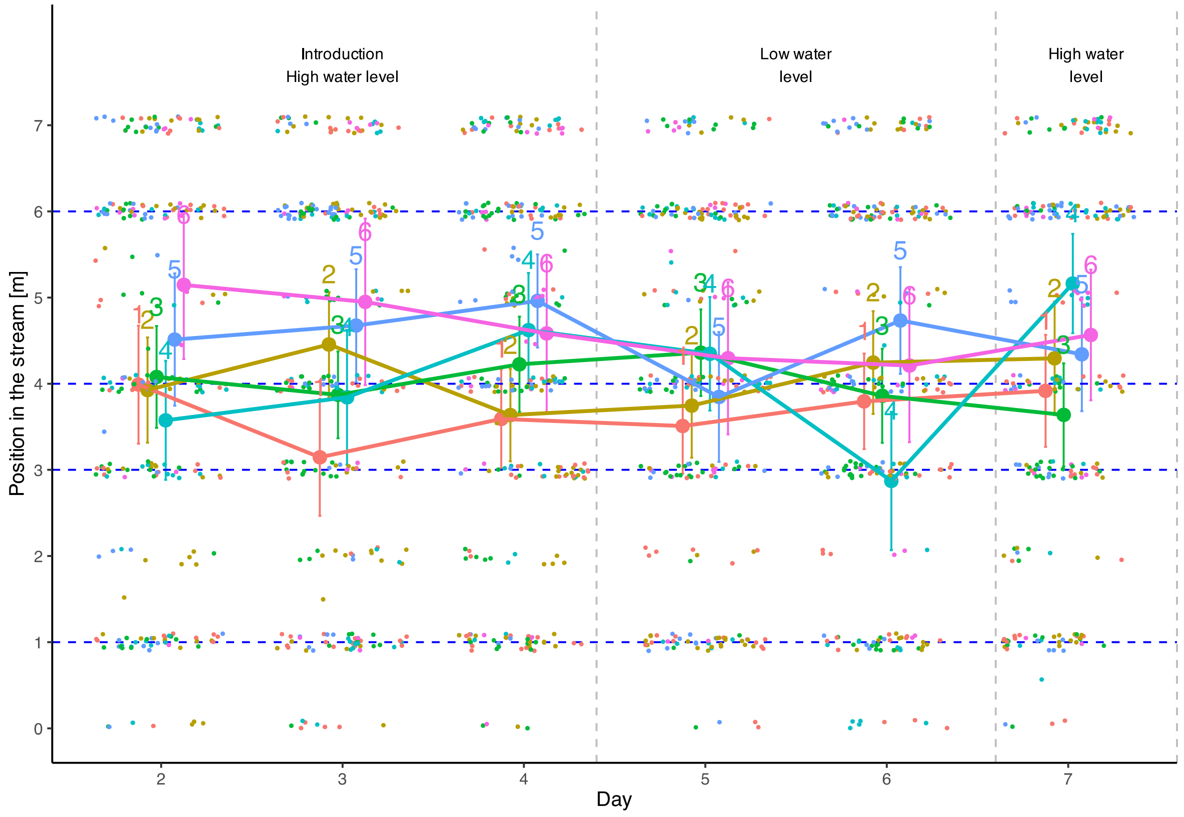

Figure S7. Error bar plot (mean ± 95% CI) showing the distribution of positions of subdominant individuals across all experimental rounds in relation to their n-6 PUFA rank (1–6) indicated by the number above the circle. Position in the stream indicates the longitudinal distance from the downstream end of each enclosure, measured in meters. Horizontal dashed lines represent the position of shelters in the enclosure, while vertical dashed lines indicate the boundaries between periods of high and low water levels.

Journal of Experimental Biology • Supplementary information

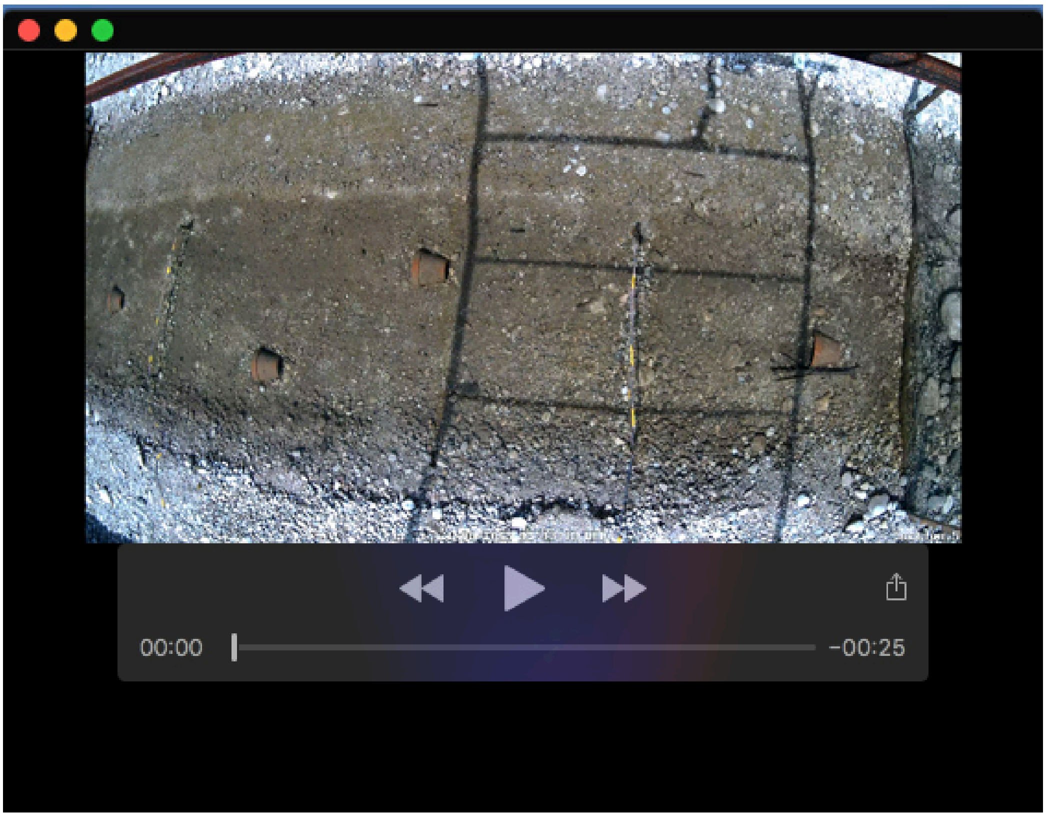

Movie 1. Sample video showing behavioural interactions of a dominant individual.

Journal of Experimental Biology • Supplementary information
